# Chloroplast Genome Variation in Upland and Lowland Switchgrass

**DOI:** 10.1371/journal.pone.0023980

**Published:** 2011-08-24

**Authors:** Hugh A. Young, Christina L. Lanzatella, Gautam Sarath, Christian M. Tobias

**Affiliations:** 1 Genomics and Gene Discovery Research Unit, United States Department of Agriculture, Agricultural Research Service, Western Regional Research Center, Albany, California, United States of America; 2 United States Department of Agriculture, Agricultural Research Service, Central-East Regional Biomass Center, Lincoln, Nebraska, United States of America; University of Melbourne, Australia

## Abstract

Switchgrass (*Panicum virgatum* L.) exists at multiple ploidies and two phenotypically distinct ecotypes. To facilitate interploidal comparisons and to understand the extent of sequence variation within existing breeding pools, two complete switchgrass chloroplast genomes were sequenced from individuals representative of the upland and lowland ecotypes. The results demonstrated a very high degree of conservation in gene content and order with other sequenced plastid genomes. The lowland ecotype reference sequence (Kanlow Lin1) was 139,677 base pairs while the upland sequence (Summer Lin2) was 139,619 base pairs. Alignments between the lowland reference sequence and short-read sequence data from existing sequence datasets identified as either upland or lowland confirmed known polymorphisms and indicated the presence of other differences. Insertions and deletions principally occurred near stretches of homopolymer simple sequence repeats in intergenic regions while most Single Nucleotide Polymorphisms (SNPs) occurred in intergenic regions and introns within the single copy portions of the genome. The polymorphism rate between upland and lowland switchgrass ecotypes was found to be similar to rates reported between chloroplast genomes of *indica* and *japonica* subspecies of rice which were believed to have diverged 0.2–0.4 million years ago.

## Introduction

Switchgrass is a warm season C_4_ perennial grass that is native to North America, occurring from Mexico to Canada, east of the Rocky Mountain Range. It is envisioned as a source of biomass for bioenergy production in marginal areas that would not compete with food production [1]–[4]. Historically, natural populations of switchgrass have been classified into two main ecotypes, upland and lowland, based on morphology and habitat [5]. Phenotypic differences exist between upland and lowland ecotypes that are reflected at the genotypic level, where substantial genetic variation exists between and within ecotypes [6]–[7].

Lowland accessions are mainly tetraploids (2n = 4x = 36) while most upland accessions are octaploid (2n = 8x = 72) [8]. Nevertheless, different ploidy levels have been shown to be common in the upland populations and these populations may also contain large numbers of aneuploid individuals [9]. In many cases, valid comparisons across ploidy levels are difficult for populations because orthologous loci are not easily identified. Gene copy number is affected by ploidy and allele frequencies within populations are affected by random pairing and assortment of chromosomes under polysomic inheritance. To circumvent these difficulties associated with nuclear loci, chloroplast (cp) genomes are often used to compare species and/or individual ecotypes. Due to the common occurrence of different ploidy series in North American grassland ecosystems, analysis of population structure using chloroplasts can contribute to a greater understanding of the dynamic evolutionary processes that have taken place during establishment of these prairie ecosystems from separate subpopulations that existed prior to the most recent glacial periods [10].

In most land plants, cp genomes consist of a single circular chromosome with a quadripartite structure that includes a large single copy region (LSC) and a small single copy region (SSC) separated by two copies of inverted repeats (IR). The gene content, order, and organization of cp genomes are generally highly conserved and inheritance is primarily maternal [11]–[12]. Such a uniparental mode of inheritance makes cp genomes invaluable for genetic and phylogenetic studies, as well as excellent substrates for genetic transformation [13]. Plastid transformation has been shown to result in high levels of transgene expression [14], the ability to co-express multiple genes [15], and a high level of transgene containment via maternal inheritance [13]. In addition, transplastomic strategies for heterologous protein expression in plants have been shown to be enhanced by customization of cp transformation vectors in a sequence-specific manner [16].

Chloroplast genetic variation between switchgrass ecotypes has been previously identified through the detection of a *BamHI* RFLP polymorphism present in *rbc*L present in upland and absent in lowland cultivars [7]. Moreover, Missaoui *et al.* identified a deletion of 49 nucleotides in *trnL-UAA* intron sequences of lowland cp genomes [17]. Phylogenetic analysis of *trnL-UAA* introns across several switchgrass accessions with unknown affiliation were able to resolve these into upland and lowland ecotypes, but bootstrap support was generally weak [17]. In addition, this 49 bp insertion/deletion (indel) was not found to be strictly diagnostic of lowland versus upland ecotypes as it was found to be present in two lowland accessions, Miami and Wabasso [18]. These earlier studies highlight the heterogeneous nature of switchgrass, and emphasize both the need and the potential for genetic markers to distinguish between genotypes. In addition, the report of heterosis for upland x lowland ecotype crosses further underscores the need for accurate and efficient discrimination of switchgrass gene pools [19], [20]. In particular, greater genetic distinction between upland and lowland ecotypes would allow for the conservation of particular germplasm, a greater understanding of cultivar diversity, and improved analyses of population structure, gene flow, and genetic mapping.

In this article, we report the complete chloroplast (cp) nucleotide sequences of two reference individuals of *Panicum virgatum* L. Our goal is to compare both an individual lowland ecotype (Kanlow) and an individual upland ecotype (Summer), with other completely sequenced grass cp genomes, and to one another. Complete cp genome alignments enabled the examination of gene content, gene order, and overall genome size. In addition, we determined the distribution and location of microsatellite repeat polymorphisms, insertions and deletions (indels), and single nucleotide polymorphisms (SNPs) among these cp genomes. Comparisons using a specific subset of protein-coding genes allowed for phylogenetic analyses of cp genomes and identified unique genetic qualifiers classifying switchgrass in the Panicoideae subfamily. Our analyses of two switchgrass cp genomes provide detailed genetic data differentiating upland and lowland ecotypes and support the utility of using plastid sequence information in breeding programs.

## Results

### Size, quality, and gene content

**Figure 1:**
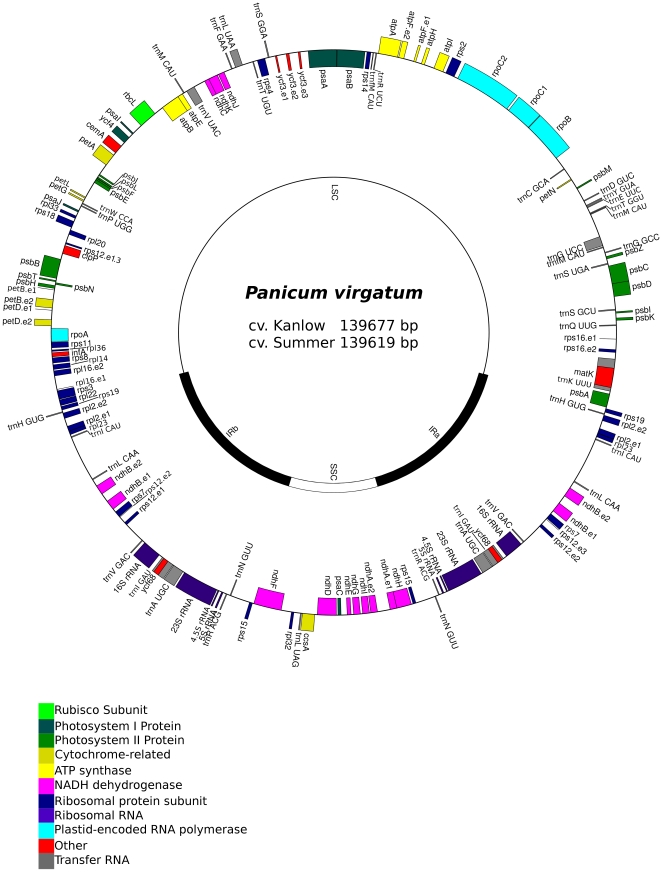
Map of the chloroplast genome of *P. virgatum* cv. Kanlow Lin1. The thick lines of the inner circle indicate the locations of the inverted repeats (IRb and IRa) which separate the SSC and LSC regions. Genes on the outside of the map are transcribed in a counter-clockwise direction and those on the inside are transcribed clockwise. Genes containing introns are marked with exon numbers (e.g. ycf3.e2). Transfer RNAs are indicated by gray bars.

The complete cp genome size of the lowland ecotype reference sequence (Kanlow Lin1) is 139,677 base pairs while the upland sequence (Summer Lin2) is 139,619 base pairs. The Kanlow Lin1 reference sequence includes a LSC region of 81,729 bp and a SSC region of 12,540 bp, which are separated by a pair of inverted repeat (IR) regions of 22,704 bp. A diagram of the Lin1 genome is represented in Figure 1. After sequence finishing and assembly with phrap, the Kanlow Lin1 and Summer Lin2 assemblies had average error rates of 0.062 and 0.005 errors per 10,000 bp, respectively, with 61 and 23 sites, respectively, below a sequence quality of phred 30. Assembled regions covered by a single Sanger read totaled 522 bp for Kanlow Lin1 and 75 bp for Summer Lin2. Assembled regions for which the consensus was determined based on reads in a single orientation totaled 6.0% and 2.5% of the genome length for Kanlow Lin1 and Summer Lin2, respectively. Each inverted repeat was assembled independently based on sequences derived from overlapping, long-range Polymerase Chain Reaction (PCR) products containing one unique primer and one repeated primer. The cp genomes for the two individuals were highly conserved and each contained a complement of 113 different genes, 19 of which were duplicated in the IR, giving a total of 132 genes (Figure 1). There were 30 unique tRNAs, 8 of which were duplicated in the IR, and 4 distinct rRNAs that were all duplicated in the IR region. Protein-coding genes comprised 43.2% of the entire cp genome and 16 of these genes contained one or more introns. Overall, the genomic GC nucleotide composition of the entire switchgrass cp genome was 38.59%. Within the inverted repeat region, the GC content was 44.01%, whereas within the LSC and SSC, the GC content was 33.10% and 36.43%, respectively. This difference was accounted for by the GC-rich nature of the four rRNAs encoded within the inverted repeat which were 55.0% GC. The tRNA genes were 52.8% GC, while the predicted protein coding sequences were 39.0% GC. Intergenic regions were 35.0% GC.

When compared to Sorghum (131 genes) [21], the difference in reported gene number for switchgrass cp (132 genes) is due to differences in annotation of *ycf68*. *Ycf68* may encode a functional protein in chloroplasts, or may be involved in the splicing of the *trnI-GAU* intron sequence [22]. A complete open reading frame is present in the two switchgrass individuals as well as Zea, Triticum, and Oryza cp genomes, while in Sorghum, there appears to be a frame-shift mutation that would preclude its function as a protein coding sequence [22], [23]. There are also two genes in the switchgrass cp genome that utilize non-ATG start codons. The *rpl2* gene utilizes GCG and the *rps19* gene utilizes GTG.

**Figure 2:**
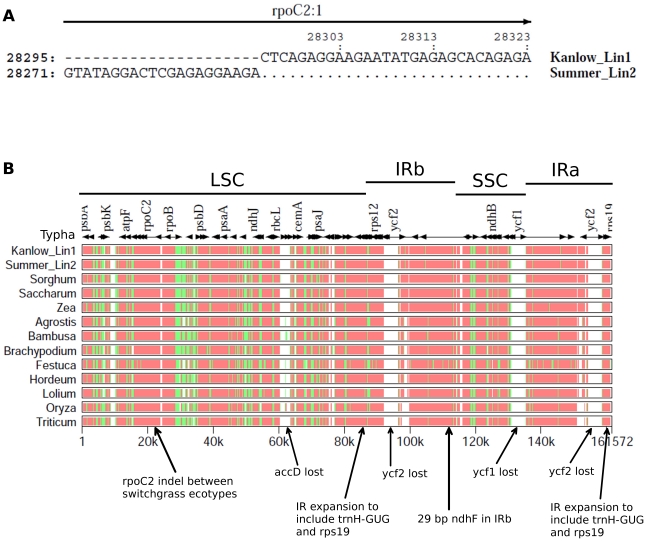
MultiPip analysis showing sequence similarity of cp genomes. (A) MultiPipMaker [26] was used to align the two switchgrass cp genomes. There is a 21 bp insertion in *rpoC2* of Summer Lin2. (B) MultiPip alignment of cp genomes from members of Poales demonstrates sequence similarity, indicated by red (75–100%), green (50–75%), and white (<50%). The earliest diverging member, *Typha latifolia*, is used as the reference genome. Arrows indicate gene losses and/or IR expansions occurring in switchgrass Lin1 and Lin2 cp genomes. Regions of the cp genome are indicated across the top (LSC, IRb, SSC, IRa).

The differences in cp genome length between the two switchgrass ecotypes, Kanlow Lin1 and Summer Lin2, were accounted for by a total of 224 bp of insertions and deletions that resulted in a 58 bp difference, overall. Insertion-deletions larger than 17 bp are shown in Table 1. A 21 bp insertion in Summer Lin2 at the C-terminal region of *rpoC2* (the beta subunit of RNA polymerase) is a key diagnostic difference between the cp genomes of these two switchgrass ecotypes (Figure 2A). These comparisons highlight the documented variability of this grass-specific, repetitive insertion sequence in the *rpoC2* gene [24], [25].

**Table 1:** Large Indels between Kanlow Lin1 and Summer Lin2 ecotypes.

Insertion	Position^1^	Length (bp)	Location	Sequence
Lowland	6248–6265	17	rps16-psbK	ACTAATAATACAACAAA
Upland	28227–28246	19	rpoC2	GTATAGGACTCGAGAGGAAGA
Upland	48626–48672	47	rps4-ndhJ	AATTAGGAATGATTATGAAATATAAAATTCTGAATTTTTTTTAGAAT
Lowland	49333–49374	42	rps4-ndhJ	TTTTCTTTCTGGTTCTTTTCTTTTTCTTTCTGGTTCTTTTCT
Lowland	53233–53264	32	ndhC-atpE	ATAATATAATATAATATAAACATACCAATAAT
Lowland	58304–58325	23	rbcL-psaI	AAAAATCCATAAAAAGTATTCTA
Lowland	63685–63709	25	psbE-petL	AATTCCTTTTTTCTCTTCTTTGTTC
Upland	107092–107108	17	ndhF-rpl32	TTAAATTTTTCCTTTTG

1 Position numbers refer to the Kanlow Lin1 cp genome.

### Genome Organization

The complete cp genomes of both switchgrass ecotypes were aligned to other members of the Poales order using MultiPipMaker [26] and the results are displayed in Figure 2B. Sequences from eleven other genera of grasses along with the early diverging Poales *Typha latifolia* were analyzed for cp genome similarity. Gene content and order were highly conserved among all grass cp genomes analyzed. Other than *ycf68*, gene order was completely conserved between the two switchgrass ecotypes and sorghum (Figure 1 and 2). Alignment of the entire Kanlow Lin1 genome with sorghum shows an overall difference in genome length of 3,157 bp, which is accounted for by length differences in the intergenic regions and introns, totaling 2,525 nt (Figure 2B and data not shown). In addition, both lowland (Lin1) and upland (Lin2) accessions of switchgrass have lost the three genes *accD*, *ycf1* and *ycf2*, which is consistent with cp genomes of other Panicoid grasses [21], [27], [28].

Expansions and contractions of the inverted repeat (IR) regions have led to variation in sequence duplication at the IR/LSC and IR/SSC boundaries of cp genomes. In the order Poales, all members have expanded these boundaries to add *trnH-GUG* and *rps19* to the IR [28]–[29]. This IRb/LSC and IRa/LSC duplication of *trnH-GUG* and *rps19* is also shared by both ecotypes of switchgrass (Figure 2B). Kanlow Lin1 and Summer Lin2 also demonstrate an expansion at the IRb/SSC boundary that has duplicated 29 bp of *ndhF*. This duplication of *ndhF* is also found in the other members of the Panicoideae, and is unique to this subfamily [28]. Unlike other genera within Poales, switchgrass does not contain an expansion of the IRa/SSC boundary that results in a partial duplication of *ndhH*. This expansion is restricted to the Ehrhartoideae and Pooideae subfamilies [28].

### Simple Sequence Repeat (SSR) Markers

**Figure 3:**
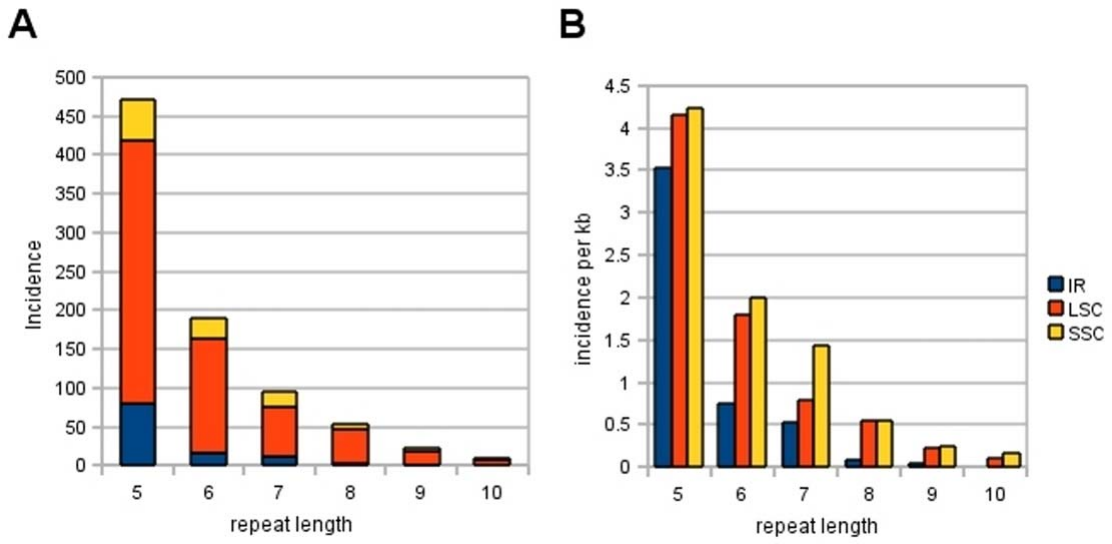
Mononucleotide microsatellite length polymorphisms in Kanlow Lin1. (A) The total incidence of mononucleotide repeats is indicated based on repeat length (bp) and location in the plastid genome. (B) The rates of homopolymer incidence per kb are indicated for each genomic region. IR – inverted repeat; LSC – long single copy; SSC – short single copy.

Mononucleotide microsatellite length polymorphisms have been used as markers in cp genomes for understanding evolutionary history due to their high rates of variability [30], [31]. Table 2 lists the positions of mononucleotide repeats of 10 bp or greater in the Kanlow Lin1 reference sequence. The numbers of mononucleotide repeats were found to be non-randomly distributed with respect to the single copy and IR regions, as well as coding and noncoding regions (Figure 3A and B). The total incidence and distribution of mononucleotide repeats is described in Figure 3A, while the rate of homopolymer size per kb is shown in Figure 3B. Overall, there were significantly more mononucleotide repeats greater than 5 bp in the single copy noncoding regions than expected, considering GC content in these regions (LSC 33.10%; SSC 36.43%). Significantly fewer repeats than expected of 6 bp or greater were found in the noncoding regions of the IR, despite the GC content (44.01%). Individually, these differences were significant for size classes of 5–9 bp for coding verses noncoding capacity and significant for repeat lengths of 6–8 bp for single copy versus IR regions (Figure 3B).

**Table 2:** Chloroplast mononucleotide microsatellites in switchgrass Lin1 of length 10 bp or greater.

Location	Sequence	SSR start^1^	SSR end
rps16-trnQ	(A/T)_10_	6150	6159
psbK-psbI	(A/T)_11_	7208	7218
trnG-trnfM	(A/T)_10_	12705	12714
trnT-trnE	(A/T)_11_	15750	15760
psbM-petN	(A/T)_10_	18609	18618
rpoC2	(A/T)_10_	30757	30766
atpF-intron	(A/T)_10_	34984	34993
psaA-ycf3	(A/T)_11_	43089	43099
ycf3-intron	(A/T)_11_	44458	44468
ycf3-intron	(A/T)_11_	45437	45447
ndhK	(A/T)_15_	51110	51124
ndhC-trnV	(A/T)_10_	51710	51719
ndhC-trnV	(A/T)_13_	52015	52027
ndhC-trnV	(A/T)_12_	52424	52435
atpB-rbcL	(A/T)_11_	55479	55489
rpl33-rps18	(A/T)_11_	66869	66879
petB-intron	(A/T)_12_	72709	72720
petD-rpoA	(A/T)_13_	75521	75533
infA	(A/T)_10_	77752	77761
InfA-rps8	(A/T)_10_	77788	77797
rpl16-intron	(A/T)_10_	79476	79485
rpl16-intron	(A/T)_13_	79578	79590
rpl16-intron	(A/T)_10_	80185	80194
ndhD-psaC	(A/T)_10_	111097	111106
trnD-psbM	(G/C)_10_	16893	16902

1 Numbering according to Lin1 genbank sequence (GenBank Acc# HQ731441).

### Insertions and Deletions (Indels)

Detailed comparisons between switchgrass ecotypes Kanlow Lin1 and Summer Lin2 have resulted in a number of descriptive polymorphisms. A total of 46 insertions and deletions were identified between the two reference sequences. These sites were located exclusively in non-coding regions with the exception of the *rpoC2* insertion (Table 3 and Figure 2A). Of these indels, 34 were associated with homopolymer repeats containing an average of 8.6 bp.

**Table 3:** Summary of polymorphisms detected between Lin1 and Lin2 chloroplast genomes.

Gene	In/Del	Tn^1^	Tv^2^	Nonsyn	Total
*atpB*		2			2
*atpF*		1			1
*ccsA*		1			1
*matK*		1	1	1	2
*ndhA*			1		1
*ndhD*			1	1	1
*ndhF*		2	1		3
*ndhH*		2			2
*ndhK*			1		1
*rbcL*		1	1	2	2
*rpl22*			1	1	1
*rpl36*		1			1
*rpoB*		1			1
*rpoC1*		1	1		2
*rpoC2*	1	2	3	4	6
*rps3*			2		2
Subtotal coding	1	15	13	9	29
Subtotal noncoding	45	26	62	-	133
Total	46	41	75	9	162

1 Tn, Transition.

2 Tv, Transversion.

Other polymorphic sites in the switchgrass cp genome have been recently assessed in the *trnT-trnL*, *atpH-atpI*, and *psbJ-petA* intergenic regions [18]. After sequencing these regions, 12 polymorphic sites were distinguished in individual cultivars. Of those differences reported by Zalapa *et al.*, 11 polymorphisms were also present in the cp reference sequences described here, while the 12^th^ was present in a different accession. In addition, comparisons between Lin1 (lowland) and Lin2 (upland) confirmed the previously described deletion of 49 nucleotides in *trnL-UAA* intron of lowland cp genomes [17].

### Single Nucleotide Polymorphisms (SNPs)

In all, there were 116 SNPs identified between the two switchgrass cp genotypes (Table 3). The substitution rate in the single copy regions was 0.00123 per nucleotide, while the rate in the inverted repeat region was 0.000088. The observed ratio (R) of transitions (Tn) to transversions (Tv) was 0.55. There were 20 synonymous substitutions in the single copy coding regions out of 10,100 possible sites, which gives an estimated substitution rate (*d*_S_) of 0.0017, based on the method of Yang & Nielsen [32]. Using the molecular clock estimates listed in Muse [33] of 2.1−2.9×10^−9^ synonymous substitutions per site per year in the cp single copy region, these sequences apparently diverged from a least common ancestor approximately 523–845 thousand years ago.

**Figure 4:**
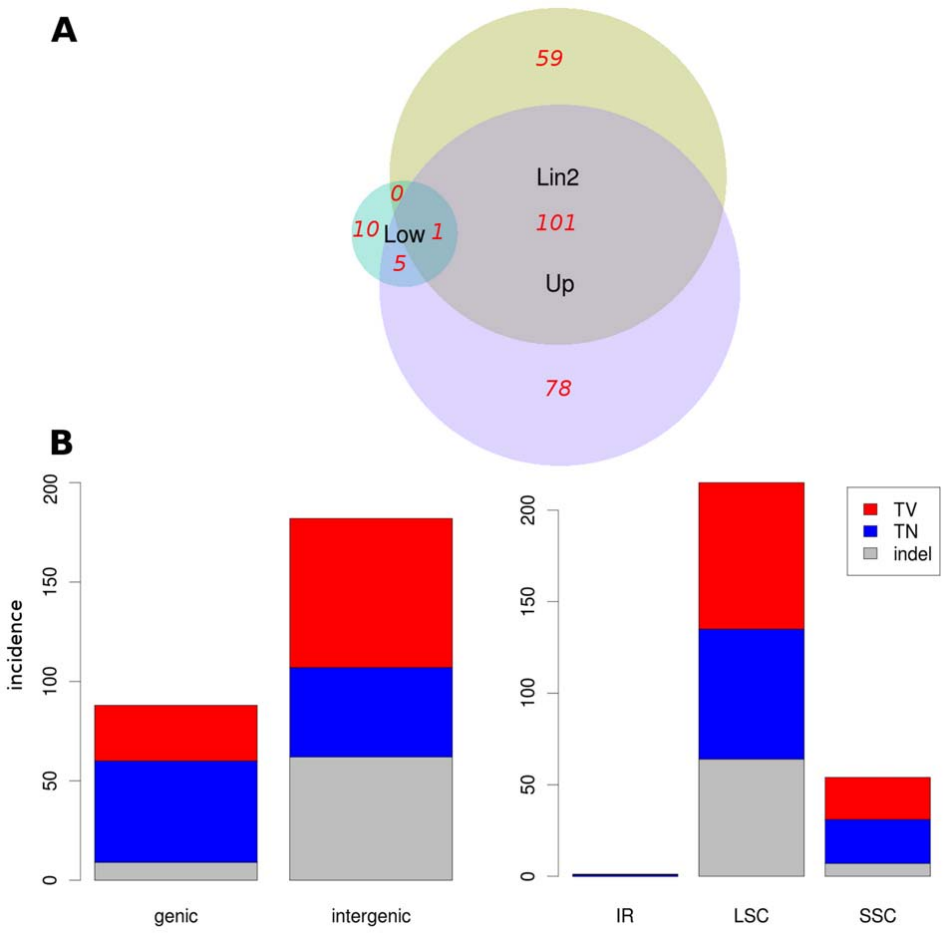
Overlap and classification of Single Nucleotide Polymorphisms (SNPs) and Insertion/Deletion (InDel) differences. Illumina RNAseq data from upland and lowland genotypes were aligned to the Lin1 reference sequence. (A) Overlap of SNPs identified within 1.53 million Illumina sequences that aligned from pooled upland genotypes (Up) and 82,656 Illumina sequences from pooled lowland (Low) genotypes with SNPs identified within the Lin2 reference genome. Numbers in red indicate the total of both variable and invariant differences that were detected. (B) The variant positions within the Illumina data were aggregated and summarized by position and by type of variation. TN, transition; TV, transversion; indel, insertion/deletion.

As an independent confirmation of the presence of these SNPs in breeding pools of switchgrass, a total of 106.5 million Illumina short-read sequences derived from RNA-seq experiments conducted on upland genotypes and 101.3 million similar sequences derived from lowland genotypes were aligned to the Lin1 reference sequence. All together, 1.53 million (1.4%) reads from upland genotypes produced at least one alignment with a maximum of 1 mismatch, and these touched 99.4% of the genome to a coverage depth of at least 4. Overall, 184 sites with a sequence depth of >4 were identified as potential SNPs/indels. Of these 184 sites, 101 (61%) matched the 116 variable positions found between the two reference sequences. When sequences derived from lowland genotypes were aligned, a total of 82,656 (0.08%) matched under the same conditions, and 90% of the genome was covered at a depth of greater than 4 reads. There were 11 variable positions and 3 indels with a sequence coverage depth of >4 within the lowland Illumina data. One additional A to T difference (pos. 18114) relative to the Lin1 sequence was invariant within the Illumina data with a coverage depth of 5. Six of the sequence differences were shared by the upland groups of reads. These data are summarized in Figure 4. Considering the variation between the reference sequences and excluding that portion that was determined to be shared among ecotypes, the total rates of inter-ecotype polymorphism were 0.07% and 0.03% for SNPs and indels, respectively. Most of this variation occurred within the non-coding regions (Figure 4B).

### RNA editing predictions

Post-translational modifications such as RNA editing can alter the amino acid sequence of a protein, causing it to differ from that predicted by the genomic DNA sequence. We find that predicted RNA editing sites occur in the switchgrass cp genome, using CURE-chloroplast v1.0 software [34]. The CURE RNA editing predictions presented in Table 4 included the C-U editing of the *rpl2* start codon to AUG. In total, there were 35 predicted editing sites, of which 28 would result in alterations to the coding sequence at non-synonymous sites. We compared the predicted editing locations with alignments of the Illumina sequencing data derived from RNA-seq experiments. All of the 35 predicted editing sites were covered by the short read sequences at a depth of at least 4. However, only two of the predicted sites (at position 1949 in *matK* and at position 78,098 in *rps8*) appeared to support editing. In addition, these reads were a minor component of the total number of aligned reads at these sites, with 2/12 and 5/13 reads consistent with editing, respectively. When considered together with the general agreement of the Illumina SNP discovery results and the reference cp genomes, these data indicate that the vast majority of aligned reads were derived from cp genomic DNA rather than cp RNA.

**Table 4:** Summary of RNA editing predicted by CURE-Chloroplast v1.0 [34].

Gene	Predicted Alteration	Lin1 coordinate
*psbA-matK*	intergenic^1^	1333
*matK*	H420Y	1949
*rps16*	intron^1^	5219
*rpoB*	S156L	21278
*rpoB*	S182L	21356
*rpoB*	P206L	21428
*rpoC2*	S904L	29017
*rpoC2*	S928L	29089
*rps2*	T45I	31316
*atpA*	S383L	37043
*rps14*	S27L	38255
*ycf3*	T20M	44666
*rbcL*	syn	56629
*rbcL*	syn	57277
*psbF*	syn	63090
*psbE*	syn	63403
*rpl20*	S103L	67672
*psbB*	A149V	70506
*petB*	P206L	73896
*rpoA*	S176F	76054
*rps8*	S61L	78098
*rpl2*	T1M	83790
*ndhB*	P494L	88687
*ndhB*	S277L	89338
*ndhB*	P246L	90141
*ndhB*	S204L	90267
*ndhB*	H196Y	90292
*ndhB*	P156L	90411
*ndhF*	S21L	106561
*ndhD*	S293L	110114
*ndhD*	intron	112248
*ndhA*	S354F	113719
*ndhA*	S185L	114226
*ndhA*	S155L	115327
*ndhA*	S14L	115750

1 intergenic and intron regions represent false positive predictions by CURE-CHLOROPLAST.

### Phylogenetic Relationships

**Figure 5:**
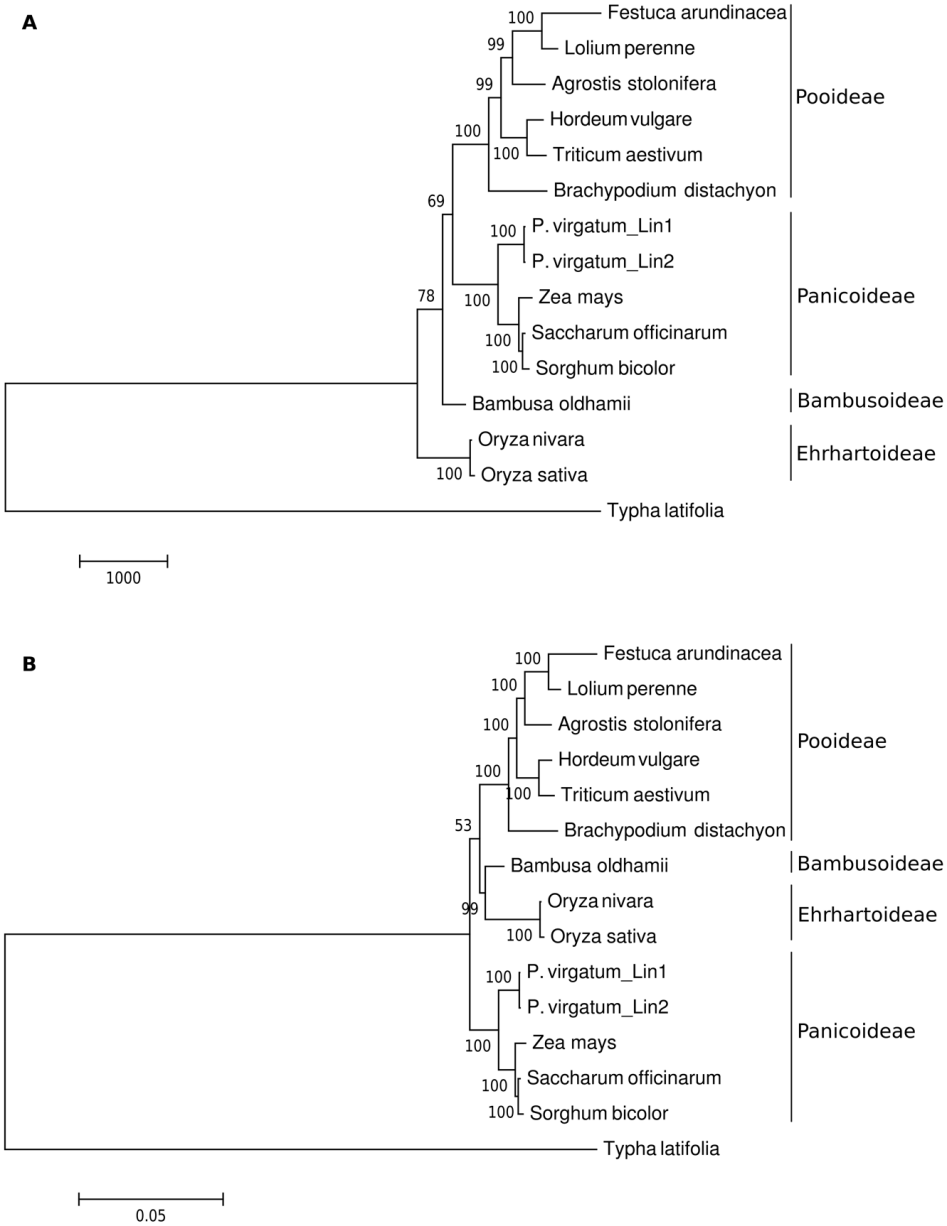
Phylogenetic Analyses. An aligned data set of 61 protein-coding genes from 15 taxa of the order Poales was used for phylogenetic analyses. The evolutionary history was inferred using the Maximum Parsimony (A) and Maximum Likelihood (B) methods. The percentage of replicate trees in which the associated taxa clustered together in the bootstrap tests (500 replicates) are shown next to the branches. There were a total of 41,397 positions in the final dataset. Evolutionary analyses were conducted in MEGA5 [67]. All positions containing gaps and missing data were eliminated. Subfamily groupings are indicated by solid lines on the right margin.

Phylogenetic analyses were performed on an aligned data set of 61 protein-coding genes [35]–[36] from 15 taxa of the order Poales (see Table S1). These monocot genera represent 4 of the 12 recognized subfamilies (sensu GPWG 2001) [37] of grasses (Bambusoideae, Ehrhartoideae, Panicoideae, and Pooideae). After gaps are excluded to avoid ambiguities in alignment, the data matrix includes a total length of 41,397 nucleotide positions. MP analysis resulted in a single most-parsimonious tree with length of 175 steps, a consistency index of 0.689 (excluding uninformative characters), and a retention index of 0.780 (Figure 5A). Bootstrap analyses (500 replicates) indicate that 10 of the 12 nodes have bootstrap values of 99–100%, giving strong support for most clades. Slightly less support is found at the node separating the Pooideae and Panicoideae subfamilies (69%) and at the node separating Bambusoideae from the other taxa (78%). Maximum Likelihood analysis resulted in a single tree with a ML value of -lnL = 134,278.23 (Figure 5B). Again, 100% bootstrap support (500 replicates) is found for all nodes of the ML tree, with two exceptions (53%, 99%). The MP and ML trees were largely similar to one another with the only differences in topology occurring at the placement of subfamilies containing Bambusa and Oryza. Phylogenetic analyses of the 61 protein-coding genes used in this study have led to grouping of *Bambusa oldhamii* with *Oryza* species in the ML tree, but support for monophyly with the Pooideae and Panicoideae subfamilies in the MP tree (Figure 5).

Both ecotypes of *Panicum virgatum* L., Kanlow Lin1 and Summer Lin2, group together with the other grass species of the Panicoideae subfamily (Figure 5). There is strong support for this grouping with 100% bootstrap values in both MP and ML trees. As expected, the two switchgrass ecotypes also group strongly with one another. Further comparisons of the inter-ecotype differences will be discussed below. Overall, monophyly of most clades was strongly supported by both the MP and ML methods. The trees described here are largely supported by recent analyses of other cp genomes [28], [38].

## Discussion

Although gene order and content among grass cp genomes are highly conserved, the differences that do exist can be highly indicative of species and subspecies variation. Our analyses of complete cp genomes from two ecotypes of switchgrass provide evidence for unique variations between the two lineages. The rates of inter-ecotype nucleotide polymorphism which we observed between switchgrass Lin1 and Lin2 are very similar to those found between *indica* and *japonica* rice cp genomes [39]. Intersubspecific polymorphism rates between rice varieties were 0.05% for SNPs and 0.02% for insertions or deletions. Our results for inter-ecotype polymorphism rates were slightly higher at 0.07% for SNPs and 0.03% for indels, indicating that insertions and deletions were less common than substitutions and that switchgrass chloroplasts are diverged to a similar extent to those of the two subspecies of *O. sativa.* Based on molecular clock estimates, these genomes diverged from a least common ancestor approximately 523,000 to 845,000 years ago [36] and generally reflect the polymorphism present in upland and lowland gene pools. However, the reference genomes clearly do not cover all the cp variation within the species as indicated by the 78 polymorphic sites that were not present in the Lin2 reference genome but which were present in the Illumina data. Moreover, the data do not provide detailed insights into the population structure that now exists within the species' natural range.

Few studies have examined variation of the cp genome within a population of a species. However, previous work has genotyped 1575 individuals of Festuca, Lolium, and Festulolium populations and discovered over 500 haplotypes [40]. Further work to sequence the entire Lolium cp genome from mixed genotypes of a single cultivar resulted in the discovery of 10 indels and 40 substitutions within this single cultivar [41]. These data are consistent with our findings of substantial variation within the switchgrass cp genome. As was successful for Lolium haplotypes [40], genotyping cp variation in switchgrass has the potential to resolve relationships between subpopulations. Variation in plastid genomes of switchgrass would not only be useful for this, but also in the expansion of cytoplasmic gene pools in breeding efforts. Plastid type variation has been used for enhancing yield gain as shown in potato [42], and for breeding of bioenergy relevant traits, as recently suggested for Miscanthus [43].

The distribution of mononucleotide repeat polymorphisms in the Kanlow Lin1 reference sequence was also similar to those described for *indica* and *japonica* rice cp genomes [39]. When taking into account GC content for single copy versus IR regions and for coding versus noncoding regions, rates of homopolymer incidence per kb did not conform to expectations. Significantly more repeat polymorphisms than expected were found in the low GC noncoding single copy regions, while significantly fewer repeats than expected were found in the noncoding regions of the IR, which contain higher GC content. A significant positive correlation exists between GC content and higher rates of recombination-associated DNA repair [44]. Moreover, research has shown that GC mutational biases are important for regulating base composition in plastid genomes [45]. In contrast, the distribution of mononucleotide repeat polymorphisms in switchgrass does not correlate with GC content. As was suggested for intersubspecific differences seen in rice varieties, these results may be attributed to a GC content bias of cp-specific DNA replication and repair systems [39]. This bias results in fewer fixed mutations and more sequence variation in regions of low GC content.

Confirmation of SNPs in switchgrass classified into upland and lowland pools showed that a greater number of sequences aligned to the cp genome from upland short-read data (1.4%) than from lowland short-read data (0.08%). We believe this was due to intrinsic differences in the source library tissue. The lowland switchgrass libraries were produced from non-green (crown) tissue sources. These tissues are known to have fewer and less well-developed plastids, in comparison to the green tissues used for libraries derived from the upland genotypes [46]. Though we cannot exclude the possibility that the short-read sequencing may be partially derived from nuclear integrated copies of the cp genome or from cp RNA, these would likely only comprise a very small percentage of the 0.08% or 1.4% of reads that aligned to the reference genomes. The amount of variation detected from the lowland pooled sample was likely lower than that in the upland pool due the presence of less sequence variation, but the smaller number of reads which were aligned and the more restricted genetic base of the population that was sampled could also have been factors. These reads were skimmed from existing data produced for other purposes and thus were not ideal for analysis of genetic diversity, but still demonstrated the prevalence of the SNPs which were identified in several distinct populations. More extensive analyses of upland and lowland sequences are necessary to determine genetic diversity between ecotypes. For example, multilocus analysis of *Oryza sativa* demonstrated that the *indica* cultivar has twice as much genetic diversity as *japonica* [47]. A similar analysis in switchgrass would be highly valuable.

Chloroplast transformation has proven to be of considerable importance to many aspects of plant biotechnology, trait introgression, and breeding programs [48]. The most prominent advantage of plastid transformation over transformation of the nuclear genome involves the ability to gain high levels of transgene expression and a large amount of the desired recombinant protein [16], [49]. In addition, cp transformation provides a strong level of biological containment due to very low rates of paternal plastid inheritance [13]. This is even more significant when considering the open pollinated nature of switchgrass. Stable populations of transplastomic individuals could be developed and monitored through controlled breeding programs of switchgrass ecotypes. Knowledge of the switchgrass cp genome sequence will allow for the design of more efficient transformation vectors [16] and would benefit biotechnological improvement strategies.

Genome-wide comparisons of the two switchgrass cp genomes with other members of Poales demonstrate conservation of monocot and grass-specific phylogenetic indicators. Earlier studies have identified several features of Poaceae plastid genomes, including three inversions, the loss of introns from genes *clpP* and *rpoC1*, and the entire loss of the three genes *accD*, *ycf1*, and *ycf2* [21], [27], [28], [50]–[56]. In addition, all members of Poales have expanded IRa/LSC and IRb/LSC boundaries to include *trnH-GUG* and *rps19* in the IR [28]. Expansions and contractions of the IR region have been well documented in angiosperm cp genomes [29], [57] but the extent of these variations at the boundaries with the single copy regions is unique among grass subfamilies. Our analysis of the switchgrass cp genome demonstrates that the IRb region has expanded to duplicate 29 bp of the *ndhF* gene, which is consistent with other members of the Panicoideae (Sorghum, Saccharum, Zea). In contrast, expansion of the IRa to duplicate a region of *ndhH* is noticeably lacking from the switchgrass plastid genome, separating it from the Ehrhartoideae and Pooideae subfamilies [28]. Our phylogenetic trees provide strong bootstrap support (100%) for the classification of *Panicum virgatum* L. plastid genomes with other Panicoideae genera and are consistent with analyses of other cp genomes [28], [38]. Future comparisons of specific gene groups would lend further support for this classification, as would greater taxon sampling of whole cp genomes. In addition, sequencing and/or re-sequencing of more switchgrass ecotypes would facilitate our understanding of interploidal variations within switchgrass, thus improving the utility of existing breeding pools. Overall, our comparisons of whole cp genomes provide detailed evidence of genetic variation between lowland and upland ecotypes that can clearly resolve classification.

## Materials and Methods

### Sequencing

Individual switchgrass genotypes were selected for cp genome sequencing that were derived from cv. “Kanlow” and “Summer” and were designated LIN1 and LIN2, respectively. DNA was isolated from immature leaves using a CTAB procedure [58]. The ‘Kanlow’ and ‘Summer’ sequence assemblies' quality was assessed by weighting the average phred score for the inverted repeat region, small single copy region and large single copy region each once. Attempts were made to resequence all bases of low quality (less than phred 30).

A PCR strategy was employed due to the highly conserved nature of cp genomes. This strategy is a compromise as it avoids the need for cp gDNA isolation, but also introduces the possibility of mistakes due to lack of fidelity of polymerases. Primers used for sequencing of the switchgrass cp genome are listed in Table S2. Both copies of the inverted repeat region were amplified and sequenced separately from overlapping, long-range PCR products amplified with the primers listed in Table S3. PCR amplifications were performed with Finnzymes Phusion High-Fidelity DNA Polymerase (New England Biolabs, Cambridge MA) following the product instructions, except total reaction volumes were 5 µl. Sequencing was performed using Big Dye Terminator v3.1 kits and an ABI3730XL automated sequencer (Applied Biosystems, Foster City CA). Sequences were deposited in Genbank under accession numbers HQ731441 and HQ822121.

### Sequence Annotation

RNA editing (C-U) sites were predicted with CURE-chloroplast v1.0 [34]. The predicted editing sites are based on a training data set of 319 C-U RNA editing sites in Arabidopsis, Rice, Maize, Tobacco, *Atropa belladonna,* Phalaenopsis, Pine, Pea, and Sugarcane.

### DOGMA annotation

Initial annotation of the *Panicum virgatum* L. cp genome was performed using DOGMA (Dual Organellar GenoMe Annotator, http://dogma.ccbb.utexas.edu/) [59]. DOGMA uses a FASTA-formatted input file to identify putative protein-coding genes by performing BLASTX searches against a custom database of published cp genomes. The input nucleotide sequence was queried in all six reading frames against amino acid sequences for all genes in the DOGMA database. Putative start and stop codons for each protein-coding gene as well as intron and exon boundaries for intron-containing genes were then checked manually. DOGMA identified both tRNAs and rRNAs through BLASTN searches against cp nucleotide databases and these were verified by the user. Manual annotation was performed using Artemis [60].

### Microsatellites and SNPs

Mononucleotide microsatellite markers were predicted using MISA [61]. A goodness of fit test was performed for mononucleotide repeats classified by region or by coding capacity based on the expectation of a random distribution proportional to the relative sizes of each region. The inverted repeat region was only counted once.

### Sequence variation

Whole genome comparisons were performed between Lin1 and Lin2 with MUMmer [62]. Primers were designed flanking insertions to score length polymorphisms between Kanlow and Summer or to score specific SNP variants using allele-specific flanking primers. PCR products are separated at 80V (constant voltage) in a 2% (w/v) agarose, TAE gel.

A total of 101.3 million Illumina GAII*x* 56-bp reads were produced from cDNA libraries of *P. virgatum* cv. ‘Kanlow’ crown and rhizome tissue prior to a killing frost. Another 106.5 million Illumina GAII 36-bp reads were downloaded from the National Center for Biotechnology Information (NCBI) sequence read archive that were annotated from a variety of upland ecotypes. These reads were aligned to the Lin1 cp reference sequence using Burrows-Wheeler Aligner [63] and Samtools [64] for SNP evaluation. Alignment and reporting conditions were set to allow a maximum of 1 mismatch per read.

No specific permits were required for the described field studies.

### Phylogenetic Analysis

A set of 61 protein-coding genes included in the analysis of several other cp genomes [21], [35], [65]were extracted from the switchgrass cp genomes using DOGMA [59]. The same 61 protein-coding genes were extracted from 13 other sequenced genomes (see Table S1) and amino acid sequences were aligned using MUSCLE [66]. After manual adjustments, nucleotide sequences of these genes were aligned by constraining them to the aligned amino acids. Phylogenetic analyses using maximum parsimony (MP) and maximum likelihood (ML) were performed with MEGA5 [67]. All gap regions were excluded during analysis to avoid alignment ambiguities. The MP tree was obtained using the Close-Neighbor-Interchange algorithm [68] with search level 1 in which the initial trees were obtained with the random addition of sequences (10 replicates). Non-parametric bootstrap analyses [69] were performed with 500 replicates. Maximum Likelihood analysis was conducted based on the Tamura-Nei model using a heuristic search for initial trees [70]. Bootstrapping was performed as for MP with 500 replicates. All three codon positions were included for both MP and ML analyses.

## Supporting Information

Table S1 **Taxa included in phylogenetic analyses with GenBank accession number and reference.** ^a^Numbers in brackets correspond to the manuscript reference list, unless indicated otherwise. ^b^Cahoon AB, Sharpe RM, Mysayphonh C, Thompson EJ, Ward AD, et al. (2010) The complete chloroplast genome of tall fescue (Lolium arundinaceum; Poaceae) and comparison of whole plastomes from the family Poaceae. Am. J. Bot. 97: 49–58. ^c^Masood SM, Nishikawa T, Fukuoka S-ichi, Njenga PK, Tsudzuki T, et al. (2004) The complete nucleotide sequence of wild rice (Oryza nivara) chloroplast genome: first genome wide comparative sequence analysis of wild and cultivated rice. Gene 340: 133–139.
(DOC)

Table S2 **Sequencing primers used in this study.** ^a^Primer sequences listed with both a “+” and “−” strand position are located in the inverted repeat region. ^b^Chloroplast genome positions listed for: *Oryza sativa*, *Osa*; *Sorghum bicolor*, *Sbi*; *Triticum aestivum*, *Tae*; *Panicum virgatum, Pvi.* Primers that do not have 100% sequence identity to a given chloroplast genome do not have positions listed for that genome. Primers with positions listed only under *Pvi* are finishing primers chosen using consed's autofinishing function.
(DOC)

Table S3 **Primers used to amplify inverted repeat (IR) region.**
(DOC)
