# Supplementary material for: Chloroplast Genome Variation in Upland and Lowland Switchgrass
Source: PLoS One. 2011 Aug 24;6(8):e23980. doi: 10.1371/journal.pone.0023980 (PMC3161095; doi:10.1371/journal.pone.0023980)
Supplement: Table S1 — Taxa included in phylogenetic analyses with GenBank accession number and reference. aNumbers in brackets correspond to the manuscript reference list, unless indicated otherwise. bCahoon AB, Sharpe RM, Mysayphonh C, Thompson EJ, Ward AD, et al. (2010) The complete chloroplast genome of tall fescue (Lolium arundinaceum; Poaceae) and comparison of whole plastomes from the family Poaceae. Am. J. Bot. 97: 49–58. cMasood SM, Nishikawa T, Fukuoka S-ichi, Njenga PK, Tsudzuki T, et al. (2004) The complete nucleotide sequence of wild rice (Oryza nivara) chloroplast genome: first genome wide comparative sequence analysis of wild and cultivated rice. Gene 340: 133–139. (DOC) [file pone.0023980.s001.doc]

Table S1. Taxa included in phylogenetic analyses with GenBank accession number and reference.

| **Taxon** | **GenBank accession number** | **Reference**^a^ |
| --- | --- | --- |
| *Agrostis stolonifera* | NC_008591 | Saski et al. 2007 [21] |
| *Bambusa oldhamii* | NC_012927 | Wu et al. 2009 [56] |
| *Brachypodium distachyon* | NC_011032 | Bortiri et al. 2008 [51] |
| *Festuca arundinacea* | NC_011713 | Cahoon et al. 2010^b^ |
| *Hordeum vulgare* | NC_008590 | Saski et al. 2007 [21] |
| *Lolium perenne* | NC_009950 | Diekmann et al 2009 [41] |
| *Oryza nivara* | NC_005973 | Masood et al 2004^c^ |
| *Oryza sativa* | NC_001320 | Hiratsuka et al. 1989 [53] |
| *Panicum virgatum cv. Kanlow Lin1* | HQ731441 | Current study |
| *Panicum virgatum cv. Summer Lin2* | HQ822121 | Current study |
| *Saccharum officinarum* | NC_006084 | Asano et al. 2004 [50] |
| *Sorghum bicolor* | NC_008602 | Saski et al. 2007 [21] |
| *Triticum aestivum* | NC_002762 | Ogihara et al. 2000 [54] |
| *Typha latifolia* | NC_013823 | Guisinger et al. 2010 [28] |
| *Zea mays* | NC_001666 | Maier et al 1995 [27] |

^a^Numbers in brackets correspond to the manuscript reference list, unless indicated otherwise.

^b^Cahoon AB, Sharpe RM, Mysayphonh C, Thompson EJ, Ward AD, et al. (2010) The complete chloroplast genome of tall fescue (Lolium arundinaceum; Poaceae) and comparison of whole plastomes from the family Poaceae. Am. J. Bot. 97: 49-58.

^c^Masood SM, Nishikawa T, Fukuoka S-ichi, Njenga PK, Tsudzuki T, et al. (2004) The complete nucleotide sequence of wild rice (Oryza nivara) chloroplast genome: first genome wide comparative sequence analysis of wild and cultivated rice. Gene 340: 133-139.
