# Supplementary material for: Chloroplast Genome Variation in Upland and Lowland Switchgrass
Source: PLoS One. 2011 Aug 24;6(8):e23980. doi: 10.1371/journal.pone.0023980 (PMC3161095; doi:10.1371/journal.pone.0023980)
Supplement: Table S2 — Sequencing primers used in this study. aPrimer sequences listed with both a “+” and “−” strand position are located in the inverted repeat region. bChloroplast genome positions listed for: Oryza sativa, Osa; Sorghum bicolor, Sbi; Triticum aestivum, Tae; Panicum virgatum, Pvi. Primers that do not have 100% sequence identity to a given chloroplast genome do not have positions listed for that genome. Primers with positions listed only under Pvi are finishing primers chosen using consed's autofinishing function. (DOC) [file pone.0023980.s002.doc]

| **Table S2. Sequencing primers used in this study.** | | | | | |  |
| --- | --- | --- | --- | --- | --- | --- |
| **OligoID** | **Sequence (5'-3')** | **Strand^a^** | **Position^b^** | | | |
|  |  |  | ***Osa*** | ***Sbi*** | ***Tae*** | ***Pvi* (Kanlow)** |
| Pvi_81339R | AAAGCCGAAGTCAGTAGGAGT | - | 80227 | 82814 | 79979 | 81339 |
| Pvi_6900F | ATCCACCCTTTCTTTTGC | + |  |  |  | 6900 |
| Pvi_30350F | ATCCCAAGGGGTACAGATCC | + | 28731 | 31797 | 29369 | 30350 |
| Pvi_115355F | ATCCCGCCATGAGAAG | + |  |  |  | 115355 |
| Pvi_131646R | ATCCTTTTGTCAACGGATGC | - | 128597 | 133264 | 128611 | 131646 |
|  |  | + | 86502 | 91205 | 86264 | 89742 |
| Pvi_65452F | ATCGATCTCCCCGCTACTG | + |  |  |  | 65452 |
| Pvi_110312F | ATCGGCAATTTGACAGC | + | 107221 | 111821 | 107267 | 110312 |
| Pvi_38034F | ATCTTGTTGCACCCGGTAAC | + | 36320 | 39447 | 36788 | 38034 |
| Pvi_117021R | ATGAGCCTCGTTTTGAGGAA | - | 113940 | 118544 |  | 117021 |
|  |  | + | 101159 | 105925 |  | 104367 |
| Pvi_9712F | ATGAGTGCTATTGGCGTAGT | + | 9743 | 10781 | 9838 | 9712 |
| Pvi_4316F | AAAGGAAATAAAATAAGGACTTTTG | + |  |  |  | 4316 |
| Pvi_128213R | ATGATCCGATCGATTGC | - | 125129 | 129809 | 125147 | 128213 |
|  |  | + | 89973 | 94663 | 89731 | 93178 |
| Pvi_67913R | ATGCGTTCATTTGCCTCAA | - | 66648 | 69372 | 66511 | 67913 |
| Pvi_85008R | ATGGACCCGAATCCTTTAGT | - | 83858 | 86463 | 83619 | 85008 |
|  |  | + | 131241 | 138006 | 131256 | 136380 |
| Pvi_134531R | ATGGATCTTCTCGATTGGA | - |  | 136159 |  | 134531 |
|  |  | + |  | 88311 |  | 86858 |
| Pvi_130055R | ATGGCAAGTGCTCTTCCTTG | - | 126989 | 131666 | 127003 | 130055 |
|  |  | + | 88110 | 92803 | 87872 | 91333 |
| Pvi_79720R | ATGGCGGAACAAACCAAA | - | 78561 | 81160 | 78361 | 79720 |
| Pvi_123317R | ATGGGATTCCAACTCAGCAC | - | 120236 | 124873 | 120408 | 123317 |
|  |  | + | 94863 | 99596 | 94467 | 98071 |
| Pvi_78260R | ATGGGCAAGGACACTATTGC | - | 77089 | 79707 | 76873 | 78260 |
| Pvi_77590R | ATGGGGGATAGGGTCAAAAT | - | 76365 | 79040 | 76205 | 77590 |
| Pvi_73004F | AAATAGGGAAGAGAAAAGTCAAG | + |  | 74455 |  | 73004 |
| Pvi_42821F | ATGTAGGTTCCAGATCCAAGT | + | 41101 | 44225 | 41567 | 42821 |
| Pvi_25206F | ATTACTCGGGGCGTTCTGTC | + | 23608 | 26656 | 24313 | 25206 |
| Pvi_80888R | ATTCCCCAATTTATTAAAGAAAA | - | 79736 | 82364 |  | 80888 |
| Pvi_88889R | ATTCTGGTGTGGATGGCAAG | - | 85649 | 90352 | 85410 | 88889 |
|  |  | + | 129450 | 134117 | 129465 | 132499 |
| Pvi_83172R | ATTGTTTGAAGCACGCACAG | - | 82045 | 84652 | 81807 | 83172 |
|  |  | + | 133054 | 139817 | 133068 | 138216 |
| Pvi_134001R | ATTTTCGCGAGAGAACCAAA | - | 130996 | 135629 | 131011 | 134001 |
|  |  | + | 84103 | 88840 | 83864 | 87387 |
| Pvi_123618R | CAAAAGGAAGGAAAATCCAT | - | 120538 | 125174 | 120704 | 123618 |
|  |  | + | 94561 | 99295 | 94171 | 97770 |
| Pvi_98077R | CAAAAGGTGCTGAGTTGGAA | - | 94869 | 99602 | 94473 | 98077 |
|  |  | + | 120230 | 124867 | 120402 | 123311 |
| Pvi_119962R | AAATAGGTGTCAAGTGGAAGTG | - | 116879 | 121512 | 117041 | 119962 |
|  |  | + | 98218 | 102955 | 97832 | 101424 |
| Pvi_4052F | CAAAATGAACTGATCCCGT | + |  |  |  | 4052 |
| Pvi_128658R | CAAACATATGCGGATCAAAT | - | 125591 | 130269 |  | 128658 |
|  |  | + | 89508 | 94200 |  | 92730 |
| Pvi_93104R | CAAAGCACGAAAGCCAGTTA | - | 89899 | 94589 | 89657 | 93104 |
|  |  | + | 125200 | 129880 | 125218 | 128284 |
| Pvi_37585F | CAAAGGTTTAGAAGACCTCTGTCC | + | 35893 | 39006 | 36346 | 37585 |
| Pvi_111998F | CAAATAGATCAGAAAATGTTACGAGA | + | 108861 | 113482 | 108939 | 111998 |
| Pvi_109141F | CAAATCCAAATTGGAAAGG | + |  |  |  | 109141 |
| Pvi_170R | CAACCGTGCTAATCTTGG | - | 164 |  |  | 170 |
| Pvi_97408R | CAAGAGCGGAGCTCTACCAA | - | 94198 | 98933 | 93809 | 97408 |
|  |  | + | 120901 | 125536 | 121066 | 123980 |
| Pvi_72922F | CAAGATAATTTCTATCAGAAAACCA | + |  | 74379 |  | 72922 |
| Pvi_51430R | CAAGCCTTATTCCTATTTTGGTA | - |  | 52900 |  | 51430 |
| Pvi_115117F | AAATTAATAAGCTGTTTTGTTTCAC | + |  | 116631 |  | 115117 |
| Pvi_6851F | CAAGGAGGTATTTTGTGTTTGG | + | 6904 | 7922 | 6975 | 6851 |
| Pvi_55909F | CAATAATGATGGATTTGGTGAA | + |  | 57346 |  | 55909 |
| Pvi_69829R | CAATATGCAATGGGTAGCTT | - |  | 71291 |  | 69829 |
| Pvi_51091F | CAATGGAATCTTTTCCTTCTT | + |  | 52564 |  | 51091 |
| Pvi_92640R | CAATTGGTCTTAGTTAGTCTTCG | - | 89418 | 94110 | 89173 | 92640 |
|  |  | + | 125678 | 130356 | 125699 | 128745 |
| Pvi_51569F | CAATTTATCCAGAACTTCTCTCTT | + |  | 53034 | 50309 | 51569 |
| Pvi_42484F | CACCGATTGCGGTACA | + | 40764 |  |  | 42484 |
| Pvi_44146F | CACCGCTGCTTAATCCCTTA | + | 42383 | 45510 | 42945 | 44146 |
| Pvi_89422R | CACGGTTTTGCATGAGAGAA | - | 86182 | 90885 | 85945 | 89422 |
|  |  | + | 128917 | 133584 | 128930 | 131966 |
| Pvi_134754R | CACTATGTATGGGATGATATGAAC | - |  | 136382 |  | 134754 |
|  |  | + |  | 88083 |  | 86630 |
| Pvi_62441F | AACAAACAAAAGAATAAATAAGGG | + |  |  |  | 62441 |
| Pvi_27239F | CACTCATGGGGATCTGGT | + |  | 28710 |  | 27239 |
| Pvi_46864F | CAGACCATAATGAAAACGCAAT | + | 45236 | 48365 | 45863 | 46864 |
| Pvi_117741R | CAGATGTATCCACAATTCATTTAGAG | - | 114658 | 119268 | 114833 | 117741 |
|  |  | + | 100435 | 105195 | 100036 | 103641 |
| Pvi_63126F | CAGCCATCGCACTGTAAAAA | + | 62028 | 64633 |  | 63126 |
| Pvi_18767F | CAGCCCAAGCGAGACTTACT | + | 17618 | 20028 | 17705 | 18767 |
| Pvi_40815R | CAGCCTAGAGCCTTGAGCAT | - | 39095 | 42219 | 39561 | 40815 |
| Pvi_56344R | CAGCTACTGCAGCCCCT | - | 54253 |  | 55068 | 56344 |
| Pvi_121298R | CAGGAAAAGCTCGAACG | - | 118212 | 122848 | 118377 | 121298 |
|  |  | + | 96890 | 101624 | 96501 | 100093 |
| Pvi_43410R | CATAAAAGGCGGCAATCTA | - |  |  |  | 43410 |
| Pvi_67185F | AACAGGCTCGTATTTTATCTTTCTT | + | 65921 | 68645 | 65844 | 67185 |
| Pvi_82842R | CATCATACGATTCATATAAGATCCA | - | 81715 | 84322 |  | 82842 |
|  |  | + | 133379 | 140142 |  | 138541 |
| Pvi_69039F | CATCCCTTTCCATGTCTTCG | + | 67760 | 70497 | 67649 | 69039 |
| Pvi_73541F | CATCGATGGTCAGCAAGTAT | + | 72298 | 74991 | 72116 | 73541 |
| Pvi_103390R | CATGAAAATTCTCAATTCTCTACGG | - | 100184 | 104944 | 99785 | 103390 |
|  |  | + | 114910 | 119520 | 115085 | 117993 |
| Pvi_84777R | CATGAGATGGTAAATGAAAACG | - |  | 86232 |  | 84777 |
|  |  | + |  | 138235 |  | 136609 |
| Pvi_108711R | CATGCATCATTAACCACTGAGA | - | 105632 | 110248 | 105706 | 108711 |
| Pvi_129251R | CATGGTAGCCTGCTCCAGTC | - | 126185 | 130862 | 126204 | 129251 |
|  |  | + | 88914 | 93607 | 88671 | 92137 |
| Pvi_20819R | CATTCCCTCATTTCCATTCC | - | 19221 | 22269 |  | 20819 |
| Pvi_39972R | CCAAAATCCTGATTCGAGTA | - |  | 41376 |  | 39972 |
| Pvi_68997R | CCAAAGCTTATGGACTTGTCG | - | 67718 | 70455 | 67607 | 68997 |
| Pvi_39041R | CCAAATTAATGCCGGATAAAAA | - | 37321 | 40445 | 37787 | 39041 |
| Pvi_30520R | CCAATAAGATTGCCCGAT | - | 28901 | 31967 | 29539 | 30520 |
| Pvi_71750R | CCAATGCTTCCATAGATTCG | - | 70483 | 73211 | 70355 | 71750 |
| Pvi_113787R | CCAGGATGAGAATGGATCAA | - |  |  |  | 113787 |
| Pvi_69005F | CCATAAGCTTTGGCTTCGTC | + | 67726 | 70463 | 67615 | 69005 |
| Pvi_64314R | CCATATGGAATAAGAGGAGAAGAAA | - | 63122 | 65722 |  | 64314 |
| Pvi_38836F | CCATCCCATCAAATAAGTGGA | + | 37116 | 40240 | 37582 | 38836 |
| Pvi_104203R | AACGTTGACGTTTTCCCAAA | - | 100994 | 105761 |  | 104203 |
|  |  | + | 114105 | 118708 |  | 117185 |
| Pvi_96233R | CCATCCCTTTCCTTTTGACA | - | 93028 | 97757 | 92778 | 96233 |
|  |  | + | 122071 | 126712 | 122097 | 125155 |
| Pvi_95255R | CCATCGTTTACGGCTAGGAC | - | 92049 | 96785 | 91802 | 95255 |
|  |  | + | 123050 | 127684 | 123073 | 126133 |
| Pvi_27428R | CCATGACGGGTACGTGTG | - | 25848 | 28899 | 26561 | 27428 |
| Pvi_94402R | CCATGCGCTTCAGATTATTA | - | 91196 | 95932 | 90949 | 94402 |
|  |  | + | 123903 | 128537 | 123926 | 126986 |
| Pvi_71032R | CCATTGAGCCTGCTCTGAAT | - | 69770 | 72494 | 69643 | 71032 |
| Pvi_10991F | CCCAACGGTTTGGACTTG | + | 11022 | 12060 | 11117 | 10991 |
| Pvi_46997F | CCCAGACGGCGTATTT | + | 45369 | 48498 | 45996 | 46997 |
| Pvi_95675R | CCCATTGTAGCACGTGTGTC | - | 92469 | 97205 | 92222 | 95675 |
|  |  | + | 122630 | 127264 | 122653 | 125713 |
| Pvi_10095F | CCCATTTCGTACCAGAAAA | + | 10126 | 11164 | 10221 | 10095 |
| Pvi_93579R | AACTTGCTATCCTCTTGCCTAA | - | 90372 | 95113 | 90128 | 93579 |
|  |  | + | 124725 | 129354 | 124745 | 127807 |
| Pvi_54916F | CCCCAAATAGTCCGATTTT | + | 52794 |  |  | 54916 |
| Pvi_126141R | CCCCAGTAGTCCTAGCCGTA | - | 123058 | 127692 | 123081 | 126141 |
|  |  | + | 92041 | 96777 | 91794 | 95247 |
| Pvi_90119R | CCCCTTTTCATCAATGGACT | - | 86881 | 91576 | 86643 | 90119 |
|  |  | + | 128218 | 132893 | 128232 | 131269 |
| Pvi_124160R | CCCGAAAAGGAGTCTATTGA | - | 121080 | 125716 | 121247 | 124160 |
|  |  | + | 94019 | 98753 | 93628 | 97228 |
| Pvi_26860F | CCCGAAAAGGGGTTG | + |  | 28331 |  | 26860 |
| Pvi_35326R | CCCGAACCAAACATGAATCT | - | 33645 | 36756 | 34106 | 35326 |
| Pvi_51362F | CCCGATTCATAACTAGAAAGC | + | 49523 | 52832 | 50107 | 51362 |
| Pvi_108924F | CCCGCATTTCCAAATTATT | + |  |  |  | 108924 |
| Pvi_16814F | CCCGTCAGTCCCGAACTA | + | 16292 | 17983 | 16125 | 16814 |
| Pvi_60090F | AAAAGAGAAACCGAAGACACA | + |  | 61606 |  | 60090 |
| Pvi_66501R | CCCGTCGATTCCTTATTGGT | - |  | 67934 |  | 66501 |
| Pvi_124557R | CCCTAATCCTCCCTTCCTGA | - | 121476 | 126113 |  | 124557 |
|  |  | + | 93623 | 98356 |  | 96831 |
| Pvi_16232R | CCCTAGGGGAAGTCGAATC | - |  |  |  | 16232 |
| Pvi_16243F | CCCTAGGGGTAGGGAGTATTA | + |  |  |  | 16243 |
| Pvi_90638R | CCCTATTGTTCCGATGGAGA | - | 87400 | 92095 | 87162 | 90638 |
|  |  | + | 127699 | 132374 | 127713 | 130750 |
| Pvi_107765F | CCCTCCATTATCCTTTATATAGAA | + |  |  |  | 107765 |
| Pvi_21210R | CCCTTATGGTCTAATTCCGAG | - |  | 22660 |  | 21210 |
| Pvi_123647R | CCCTTCAACCCTTTGAGC | - | 120567 | 125203 | 120733 | 123647 |
|  |  | + | 94534 | 99268 | 94144 | 97743 |
| Pvi_138704R | CCCTTCCACGTGTGATTTCT | - | 133542 | 140305 |  | 138704 |
|  |  | + | 81557 | 84164 |  | 82684 |
| Pvi_53119F | CCCTTGTCAGAGATATCTTAACAT | + |  | 54569 |  | 53119 |
| Pvi_60217R | AAGAACCACTTAGAATAGCCAAACA | - | 59022 | 61733 | 59042 | 60217 |
| Pvi_69042F | CCCTTTCCATGTCTTCGG | + | 67763 | 70500 | 67652 | 69042 |
| Pvi_115436R | CCCTTTGGGATATCGTTTTG | - |  | 116950 |  | 115436 |
| Pvi_61165F | CCGACCGTATTTCTCCTGA | + | 59962 | 62670 |  | 61165 |
| Pvi_27367F | CCGACCTTGTACGATCCC | + | 25787 |  | 26500 | 27367 |
| Pvi_111215R | CCGAGAACCGAAGATTGTGT | - |  | 112720 | 108166 | 111215 |
| Pvi_22691R | CCGAGGTTTTTGGTGCATAC | - | 21093 | 24141 | 21798 | 22691 |
| Pvi_109967F | CCGATAAATCCATGGGATA | + |  |  |  | 109967 |
| Pvi_137375R | CCGATTCGACATTAAAAGTATATTGA | - | 132213 | 198449 | 188924 | 137375 |
|  |  | + | 82880 | 85487 | 82642 | 84007 |
| Pvi_53364R | CCGCCGTATGAAAGCAATAC | - | 51242 | 54812 | 52057 | 53364 |
| Pvi_101311R | CCGGAGGCTCTAGGGAA | - | 98105 | 102842 | 97719 | 101311 |
|  |  | + | 116997 | 121630 | 117159 | 120080 |
| Pvi_69642F | AAGAATCCACAGTTTATTATCTTCA | + |  |  |  | 69642 |
| Pvi_75038R | CCGGTTCACCAATCATTGAC | - | 73804 | 76482 |  | 75038 |
| Pvi_18055F | CCGGTTGGAATCATAAAGTG | + | 16867 |  |  | 18055 |
| Pvi_165R | CCGTGCTAATCTTGGTATGG | - | 159 | 690 |  | 165 |
| Pvi_92021R | CCGTGTCAACAAACAATTCG | - | 88798 | 93491 | 88555 | 92021 |
|  |  | + | 126301 | 130978 | 126320 | 129367 |
| Pvi_123370R | CCGTTCCTGGTTCTCCTGTA | - | 120289 | 124926 | 120461 | 123370 |
|  |  | + | 94810 | 99543 | 94414 | 98018 |
| Pvi_105011F | CCGTTTCATGAGGATAGACAAA | + |  | 106569 |  | 105011 |
| Pvi_42808R | CCTACATGCTGATGCTCACG | - | 41088 | 44212 | 41554 | 42808 |
| Pvi_112816F | CCTAGTAGAAGAACAAATCCCC | + | 109693 | 114319 | 109774 | 112816 |
| Pvi_59362F | CCTCGTCGTATCCTTTATATGG | + | 58113 | 60869 |  | 59362 |
| Pvi_49152F | CCTCGTGTCACCAGTTCAAA | + | 47464 | 50670 | 48095 | 49152 |
| Pvi_66395F | AAGAATTAAGTTAAGTCGATCCAA | + |  | 67828 |  | 66395 |
| Pvi_48828F | CCTCTATCCCCAAACCCTCT | + | 47171 | 50255 |  | 48828 |
| Pvi_105376R | CCTCTTGCTTGCTTCTGGTC | - | 102391 | 106934 | 102093 | 105376 |
| Pvi_91632R | CCTCTTTCACGCTCATGTCA | - | 88409 | 93102 | 88171 | 91632 |
|  |  | + | 126690 | 131367 | 126704 | 129756 |
| Pvi_16993R | CCTTATCAACCAGAAGTAGTGAAAA | - |  |  |  | 16993 |
| Pvi_37114R | CCTTGCCAATTGATTCTGACT | - | 35428 | 38537 | 35887 | 37114 |
| Pvi_28789R | CCTTTAGGGATGAGGATCTCG | - | 27143 | 30230 |  | 28789 |
| Pvi_58483R | CGAAAATAGAGGGTAAGTTAAAATC | - | 57228 |  |  | 58483 |
| Pvi_42802F | CGAAATCGTGAGCATCAGC | + | 41082 | 44206 | 41548 | 42802 |
| Pvi_65036F | CGAACAAATCGAAATGATTGAA | + | 63786 | 66454 | 63603 | 65036 |
| Pvi_3426R | AAGAGCGATTGGCCTG | - |  | 3953 |  | 3426 |
| Pvi_46123R | CGAACCCTCGGTAAACAAAA | - | 44489 | 47623 | 45090 | 46123 |
| Pvi_72808R | CGAATAAGAACCGCTTGATGA | - | 71551 | 74265 |  | 72808 |
| Pvi_63263R | CGACCAAACGAGTATTTCAC | - |  | 64770 |  | 63263 |
| Pvi_12281F | CGACCCAGACATAGACGG | + | 12274 | 13352 |  | 12281 |
| Pvi_52794F | CGAGACTTCATACACCTTAAAGTTC | + |  |  |  | 52794 |
| Pvi_112357F | CGAGCCATAGTAATTGCACCT | + | 109234 | 113860 | 109315 | 112357 |
| Pvi_136467R | CGAGGGCTTCTTATTCCACA | - | 131333 | 138093 | 131348 | 136467 |
|  |  | + | 83766 | 86376 | 83527 | 84921 |
| Pvi_23079R | CGATATACGACGATTCGCTC | - |  | 24529 |  | 23079 |
| Pvi_33254F | CGATATATGGAATTCCCTTCTA | + |  |  |  | 33254 |
| Pvi_43820R | AAGATAGCAATTTCTGAGCCGTA | - | 42064 | 45184 |  | 43820 |
| Pvi_74410R | CGATGCAAAGAAAATGAATGC | - | 73167 | 75854 | 72984 | 74410 |
| Pvi_27075F | CGATGCATTGCTGCTC | + |  |  |  | 27075 |
| Pvi_116493F | CGATGCGAAAGTAATTATGC | + | 113409 | 118016 |  | 116493 |
| Pvi_112176F | CGATTCATTTCAATCTGAGGA | + |  |  |  | 112176 |
| Pvi_125828R | CGCAACCCTCGTGTTTAGTT | - | 122745 | 127379 | 122768 | 125828 |
|  |  | + | 92354 | 97090 | 92107 | 95560 |
| Pvi_54202R | CGCAATTCTTGGGTTGG | - | 52080 |  |  | 54202 |
| Pvi_111122R | CGCAGCATGGCTCTATC | - | 108027 | 112627 |  | 111122 |
| Pvi_124686R | CGCATCTTCACAGACCAAGA | - | 121604 | 126242 | 121628 | 124686 |
|  |  | + | 93495 | 98227 | 93247 | 96702 |
| Pvi_29657R | CGCCTAAGAGCCCAAAA | - |  | 31104 |  | 29657 |
| Pvi_17425F | AAGATCCCCTTTTCTCCATCTC | + |  |  |  | 17425 |
| Pvi_4183R | CGCTCAAATGATAGGGTTGC | - |  |  |  | 4183 |
| Pvi_114453F | CGCTTCCGAATTGATCTCAT | + |  | 115957 | 111490 | 114453 |
| Pvi_35728R | CGGAAATGTAACTCGGTATTCA | - | 34047 | 37158 | 34504 | 35728 |
| Pvi_67965F | CGGAACTCTGGTCATTGAATC | + | 66700 | 69424 | 66563 | 67965 |
| Pvi_54841F | CGGATACGCCTCCATGA | + |  | 56289 |  | 54841 |
| Pvi_96550R | CGGATCCCACGAGTGAATAG | - | 93345 | 98074 | 93095 | 96550 |
|  |  | + | 121754 | 126395 | 121780 | 124838 |
| Pvi_26767F | CGGATCCTCAAGGACAAATG | + | 25187 | 28238 |  | 26767 |
| Pvi_129796R | CGGATTTTGCGGTTCTT | - |  | 131407 |  | 129796 |
|  |  | + |  | 93065 |  | 91595 |
| Pvi_107575F | CGGCAATGAGCATCCA | + |  |  |  | 107575 |
| Pvi_123815R | AAGATGGGCTGTCAAAAAGG | - | 120736 | 125371 | 120901 | 123815 |
|  |  | + | 94363 | 99098 | 93974 | 97573 |
| Pvi_62780F | CGGCCATTTCCCCTACT | + | 61681 | 64287 |  | 62780 |
| Pvi_34156R | CGGGAAGGAAGAGGGC | - |  | 35584 |  | 34156 |
| Pvi_81925R | CGGGCATCTAGCATTCTACC | - | 80797 | 83405 | 80559 | 81925 |
|  |  | + | 134302 | 310 | 134316 | 139463 |
| Pvi_125083R | CGGGCTATTAGCTCAGTGGTA | - | 121999 | 126640 | 122025 | 125083 |
|  |  | + | 93099 | 97828 | 92849 | 96304 |
| Pvi_122891R | CGGGGAGTTGAAAATAAGCA | - | 119809 | 124446 | 119975 | 122891 |
|  |  | + | 95290 | 100023 | 94900 | 98497 |
| Pvi_16763R | CGGTGCTCTGACCAATTGAA | - | 16241 | 17932 | 16074 | 16763 |
| Pvi_15641F | CGGTTCAAATCCGATAAAGG | + | 15109 | 16808 | 15085 | 15641 |
| Pvi_13308F | CGGTTCTAGTAAACCAAAACCA | + |  |  |  | 13308 |
| Pvi_102116R | CGTAGCCACGTGCTCTAATC | - | 98908 | 103647 | 98494 | 102116 |
|  |  | + | 116191 | 120822 | 116381 | 119272 |
| Pvi_108608F | AAGCACAATAACTACGCCAAG | + |  | 110145 | 105603 | 108608 |
| Pvi_1683R | CGTATCAATGACCTGGTGAAT | - |  | 2212 |  | 1683 |
| Pvi_96737R | CGTCACCCCAGAATGAAAG | - | 93529 | 98262 | 93282 | 96737 |
|  |  | + | 121571 | 126208 | 121594 | 124652 |
| Pvi_61658F | CGTCCAAGGCCTTTTGT | + |  | 63163 |  | 61658 |
| Pvi_27717F | CGTCGATTACCAAAAACAAGC | + | 26137 | 29188 |  | 27717 |
| Pvi_4974R | CGTCTCAACAATAGAATTAGATCAG | - |  | 5526 |  | 4974 |
| Pvi_10255R | CGTGATAAATGCCACCGAAG | - | 10286 | 11324 | 10381 | 10255 |
| Pvi_1472F | CGTGCTTGCATTTTTCATTG | + | 1461 | 1997 | 1473 | 1472 |
| Pvi_130802R | CGTTGAAATTGTTCGTTTGG | - | 127751 | 132426 | 127765 | 130802 |
|  |  | + | 87348 | 92043 | 87110 | 90586 |
| Pvi_82795R | AAGCCGTATGCTTTGGA | - | 81668 | 84275 | 81430 | 82795 |
|  |  | + | 133434 | 140197 | 133448 | 138596 |
| Pvi_10898R | CGTTGGGGAACGCATTAG | - | 10929 | 11967 |  | 10898 |
| Pvi_66044F | CGTTTATTCCCAGATGCTTT | + |  | 67466 | 64592 | 66044 |
| Pvi_9907F | CTACCACGTGGAAACGCTCT | + | 9938 | 10976 | 10033 | 9907 |
| Pvi_53394R | CTACCTACCTACTATTGGATTTGAA | - | 51272 | 54842 |  | 53394 |
| Pvi_85695R | CTAGCAGTTCCGGTTTGAC | - |  | 87149 |  | 85695 |
|  |  | + |  | 137321 |  | 135694 |
| Pvi_88936R | CTAGGAGGCCTTCCTCCACT | - | 85696 | 90399 | 85457 | 88936 |
|  |  | + | 129403 | 134070 | 129418 | 132452 |
| Pvi_129575R | CTATAGGGATCTATGAGATCGAGTA | - | 126509 | 131186 | 126528 | 129575 |
|  |  | + | 88585 | 93278 | 88342 | 91808 |
| Pvi_98861R | CTATCGGTCACCCAGGAGTATT | - | 95654 | 100387 | 95264 | 98861 |
|  |  | + | 119443 | 124080 | 119609 | 122525 |
| Pvi_130490R | CTCCGAATGTTTCGATTTT | - |  |  |  | 130490 |
|  |  | + |  |  |  | 90899 |
| Pvi_112922F | CTGACTAAAACTAAAAGATTCAAGG | + | 109799 | 114425 |  | 112922 |
| Pvi_43408R | AAAAGGCGGCAATCTAAA | - |  |  |  | 43408 |
| Pvi_2792F | AAGGAAATATTGAATGAATAGATTG | + |  |  |  | 2792 |
| Pvi_4061F | CTGATCCCGTTAAATTTCACTA | + |  |  |  | 4061 |
| Pvi_9614F | CTGCTAACCGCTTTTGG | + |  | 10683 | 9740 | 9614 |
| Pvi_33791F | CTGCTGCAGGACAAGCTGTA | + | 32127 | 35204 | 32591 | 33791 |
| Pvi_99491R | CTGGCTGTCTTTGCACC | - | 96289 | 101022 | 95899 | 99491 |
|  |  | + | 118813 | 123450 | 118979 | 121900 |
| Pvi_126076R | CTGTAGCTAACGCGTTAAGTATC | - | 122993 | 127627 | 123016 | 126076 |
|  |  | + | 92103 | 96839 | 91856 | 95309 |
| Pvi_65117R | CTGTATTGCAAATACGCAGTC | - | 63867 | 66535 | 63684 | 65117 |
| Pvi_16785F | CTGTCAAGGCGGAAGCTG | + | 16263 | 17954 | 16096 | 16785 |
| Pvi_9275F | CTGTTCAATTGCGGCCTTAT | + | 9306 | 10344 | 9401 | 9275 |
| Pvi_100297R | CTTACGACTTTGCGGAGACC | - | 97094 | 101828 | 96705 | 100297 |
|  |  | + | 118005 | 122641 | 118170 | 121091 |
| Pvi_6459R | CTTATGAACTCGTGGATTAGATG | - |  |  |  | 6459 |
| Pvi_63625R | AAGGAATTCTGTTTCGGG | - |  | 65128 |  | 63625 |
| Pvi_56293F | CTTGGCAGCATTCCGAGTA | + | 54202 | 57800 | 55017 | 56293 |
| Pvi_65536R | CTTGGTAGCGCGTTTGTTTT | - | 64268 | 66955 | 64102 | 65536 |
| Pvi_100551R | CTTTCTGTCCAGGTGCAGGT | - | 97347 | 102082 | 96959 | 100551 |
|  |  | + | 117752 | 122387 | 117916 | 120837 |
| Pvi_84792R | GAAAAGGGTTTTCCATGAGA | - |  | 86247 |  | 84792 |
|  |  | + |  | 138222 |  | 136596 |
| Pvi_131099R | GAAACCATGAACCAGAATAGAAGAG | - | 128048 | 132723 | 128062 | 131099 |
|  |  | + | 87046 | 91741 | 86808 | 90284 |
| Pvi_3315F | GAAAGAAAGGGAGTCTAATCCA | + |  | 3846 |  | 3315 |
| Pvi_66621F | GAAATAAAGAAATAGGAGCATCG | + |  | 68054 |  | 66621 |
| Pvi_23878F | GAAATACTTAATGCTACGATCTGGG | + |  | 25328 | 22985 | 23878 |
| Pvi_42298F | GAAATTGGTTAATCGGTAAAGATA | + | 40578 | 43702 |  | 42298 |
| Pvi_51142R | AAGGAGCCTTGGAATGGTCT | - | 49303 | 52612 | 49887 | 51142 |
| Pvi_109545F | GAACAAAATCTGGATAAATACCAA | + |  | 111054 | 106500 | 109545 |
| Pvi_130741R | GAACAATAGGGCCGTTATGC | - | 127690 | 132365 | 127704 | 130741 |
|  |  | + | 87409 | 92104 | 87171 | 90647 |
| Pvi_50738F | GAACATTCCGCCTGTAATAGTA | + |  |  |  | 50738 |
| Pvi_56437R | GAACGGGCTCGATGTGATAG | - |  | 57944 |  | 56437 |
| Pvi_43765R | GAACTGCCTATTCCGACC | - |  | 45129 |  | 43765 |
| Pvi_88511R | GAACTTATAATCATGGAATCGACT | - | 85271 | 89974 | 85032 | 88511 |
|  |  | + | 129824 | 134491 | 129839 | 132873 |
| Pvi_14927F | GAAGAGCACAAATTCCTTTATACT | + |  |  |  | 14927 |
| Pvi_69500R | GAAGCGACTTGGGTTGACTT | - | 68221 | 70958 | 68110 | 69500 |
| Pvi_96827R | GAAGGGAGGATTAGGGAAGT | - | 93619 | 98352 |  | 96827 |
|  |  | + | 121480 | 126117 |  | 124561 |
| Pvi_117090R | GAAGTAATTTAATCGTTCGAATTTT | - |  | 118613 |  | 117090 |
|  |  | + |  | 105851 |  | 104293 |
| Pvi_5859F | AAGGATCGACTCTTCCCC | + |  |  |  | 5859 |
| Pvi_137385R | GAATCCCGATTCGACATTAAA | - | 132223 | 198469 | 188944 | 137385 |
|  |  | + | 82875 | 85482 | 82637 | 84002 |
| Pvi_111615F | GAATTAGCCAATAAACTCGGTAT | + |  |  |  | 111615 |
| Pvi_26651F | GAATTCGAATTTTCGAATAACG | + |  | 28122 |  | 26651 |
| Pvi_51529R | GAATTGATTAATTCAGTTCCGA | - |  |  |  | 51529 |
| Pvi_71184F | GAATTGTGAGAGCGGACGTT | + | 69922 | 72646 |  | 71184 |
| Pvi_5063F | GACATTACTTCGGGAATTCCTATT | + |  |  |  | 5063 |
| Pvi_119977R | GACGAGCCGTTTAAATAGG | - | 116894 | 121527 | 117056 | 119977 |
|  |  | + | 98206 | 102943 | 97820 | 101412 |
| Pvi_40635F | GACGAGTAGTGGGGTCCTGA | + | 38915 | 42039 | 39381 | 40635 |
| Pvi_118001R | GACGCCGTAGAGAATTGAGAA | - | 114918 | 119528 | 115093 | 118001 |
|  |  | + | 100180 | 104940 | 99781 | 103386 |
| Pvi_24190F | AAGGCCCTCCACTTTTCATT | + | 22592 | 25640 |  | 24190 |
| Pvi_45700F | GAGAGGTATGGGTTTTCCG | + |  |  |  | 45700 |
| Pvi_62479F | GAGCAAAGGTTTTCTCTTTGT | + |  | 63986 |  | 62479 |
| Pvi_7907F | GAGGCAAGAAATAACGATTGC | + | 7951 | 8979 |  | 7907 |
| Pvi_47373R | GAGGGTCATCGGTTCAAATC | - | 45727 | 48876 | 46356 | 47373 |
| Pvi_55902F | GAGTATACAATAATGATGGATTTGG | + |  | 57339 |  | 55902 |
| Pvi_131997R | GAGTCAGAGTCGAAAAGAGGAT | - | 128948 | 133615 |  | 131997 |
|  |  | + | 86149 | 90852 |  | 89389 |
| Pvi_97342R | GAGTCAGATGCTTCTTCTATTCTT | - | 94133 | 98867 | 93742 | 97342 |
|  |  | + | 120962 | 125598 | 121129 | 124042 |
| Pvi_F | GAGTGGTCTTATTCAAATTCAAAGC | + | 41843 | 44963 |  |  |
| Pvi_94377R | GAGTTCGCCACCAGCAGTAT | - | 91171 | 95907 | 90924 | 94377 |
|  |  | + | 123928 | 128562 | 123951 | 127011 |
| Pvi_5862F | GATCGACTCTTCCCCAACAA | + |  |  |  | 5862 |
| Pvi_68229R | AAGGGCTCCGGTGTATAGAGA | - | 66950 | 69688 | 66837 | 68229 |
| Pvi_2892F | GATCGCAAACCCCTCAGATA | + | 2889 |  |  | 2892 |
| Pvi_56792F | GATGAAAACGTAAACTCACAACC | + | 54701 | 58299 | 55516 | 56792 |
| Pvi_28413F | GATGAATATGGGATCTTAGAGGA | + |  |  |  | 28413 |
| Pvi_14453R | GATTCTTAGGAGGAACGTTTG | - |  | 15592 |  | 14453 |
| Pvi_32396F | GCAAAACTCAGATTGGAGAAGAA | + | 30748 | 33864 | 31435 | 32396 |
| Pvi_5400F | GCAAATTCGGTTCGGG | + |  | 5955 |  | 5400 |
| Pvi_104124R | GCAAGTCTCCGTATCTTATTGG | - | 100915 | 105682 | 100524 | 104124 |
|  |  | + | 114182 | 118785 | 114349 | 117262 |
| Pvi_117582R | GCAATAAATCAGCAAAATTCTTT | - |  |  |  | 117582 |
|  |  | + |  |  |  | 103803 |
| Pvi_44580R | GCAATGGAGAGCATACAAAGG | - | 42822 | 45943 | 43382 | 44580 |
| Pvi_98700R | GCACAACGCTTGTATTGCTC | - | 95493 | 100226 | 95103 | 98700 |
|  |  | + | 119606 | 124243 | 119772 | 122688 |
| Pvi_17778R | AAGGGGAGGACTTGCATTCT | - | 16596 | 18957 |  | 17778 |
| Pvi_102771R | GCACAATTTCTCGATGTTTCG | - | 99560 | 104302 | 99169 | 102771 |
|  |  | + | 115538 | 120166 | 115705 | 118616 |
| Pvi_111052F | GCACAGGCTTCTTCGGTAAA | + | 107961 | 112561 | 108007 | 111052 |
| Pvi_83671R | GCACCGTTGTGGTAAAGGTC | - | 82544 | 85151 | 82306 | 83671 |
|  |  | + | 132555 | 139318 | 132569 | 137717 |
| Pvi_48431R | GCACCTATCCTTTTCCTTTGG | - | 46726 | 49898 |  | 48431 |
| Pvi_69075R | GCACTAAGAACAGGCAAGC | - | 67796 | 70533 |  | 69075 |
| Pvi_69176F | GCACTAGCAGGTTGGTGAAG | + | 67897 | 70634 | 67786 | 69176 |
| Pvi_108298R | GCATAAGGATAAGGTCCCATT | - |  |  |  | 108298 |
| Pvi_36192R | AAGTAAGCCTCGCTCACG | - | 34506 | 37615 |  | 36192 |
| Pvi_95648R | GCATAAGGGGCATGATGACT | - | 92442 | 97178 | 92195 | 95648 |
|  |  | + | 122657 | 127291 | 122680 | 125740 |
| Pvi_57280R | GCATGGATACCCAGTCCTGA | - | 55189 | 58787 | 56004 | 57280 |
| Pvi_47393F | GCATTACAAATGCGATGCTC | + | 45747 | 48896 | 46376 | 47393 |
| Pvi_75607F | GCCAGAGTGTCCCATATCAGT | + |  |  |  | 75607 |
| Pvi_31723F | GCCATTTTGGGGATTCCTAC | + | 30080 | 33196 | 30745 | 31723 |
| Pvi_131076R | GCCCCACCCATGAGTAAATA | - | 128025 | 132700 | 128039 | 131076 |
|  |  | + | 87074 | 91769 | 86836 | 90312 |
| Pvi_10491F | GCCCCGGTGTTATATTTGGT | + | 10522 | 11560 |  | 10491 |
| Pvi_126193R | GCCGACACTGACACTGAGAG | - | 123110 | 127744 | 123133 | 126193 |
|  |  | + | 91989 | 96725 | 91742 | 95195 |
| Pvi_75035F | GCCGTCAATGATTGGTGAAC | + | 73801 | 76479 |  | 75035 |
| Pvi_73490R | GCCTCCGTCATTATATATTGAAC | - |  | 74940 |  | 73490 |
| Pvi_117523R | AATAAAAGAATGTACCCTTCTATCC | - | 114436 | 119046 |  | 117523 |
|  |  | + | 100658 | 105418 |  | 103860 |
| Pvi_12846F | GCCTCGTGAGCTACCAAACT | + | 12880 | 13917 | 13044 | 12846 |
| Pvi_1108R | GCGAAAGTACAAGCCTGTGG | - | 1102 | 1633 | 1111 | 1108 |
| Pvi_69098R | GCGAAATGATCACAAGGGT | - | 67819 | 70556 | 67708 | 69098 |
| Pvi_139545R | GCGACCTACCATAGGATTTG | - | 134384 | 392 | 134398 | 139545 |
|  |  | + | 80715 | 83323 | 80477 | 81843 |
| Pvi_2279F | GCGAGAAATTGACAAGGTAAGA | + |  | 2814 |  | 2279 |
| Pvi_51303F | GCGCAAACATGTAATAGCGTA | + | 49464 | 52773 |  | 51303 |
| Pvi_97164R | GCGCTCTTCAAGTGTGCT | - | 93955 | 98689 | 93564 | 97164 |
|  |  | + | 121146 | 125782 | 121313 | 124226 |
| Pvi_134796R | GCGGAAACATGGAAGAAT | - |  | 136424 |  | 134796 |
|  |  | + |  | 88047 |  | 86594 |
| Pvi_97403R | GCGGAGCTCTACCAACTGAG | - | 94193 | 98928 | 93804 | 97403 |
|  |  | + | 120906 | 125541 | 121071 | 123985 |
| Pvi_45797F | GCGTAACGTACCTTATGCAA | + |  |  |  | 45797 |
| Pvi_90942R | AATAAGGTTTGATCCTATTCATGG | - | 87719 | 92409 | 87481 | 90942 |
|  |  | + | 127376 | 132056 | 127390 | 130442 |
| Pvi_46861R | GCGTTTTCATTATGGTCTGACA | - | 45233 | 48362 | 45860 | 46861 |
| Pvi_11135R | GCTAACCAACTTCTAGGAGAGAC | - |  | 12204 |  | 11135 |
| Pvi_62250F | GCTAGTTGAGTAGTTTTGATTAAGG | + |  | 63746 |  | 62250 |
| Pvi_494R | GCTATGCATGGTTCCTTGGT | - | 488 | 1019 | 497 | 494 |
| Pvi_40817F | GCTCAAGGCTCTAGGCTGAGTA | + | 39097 | 42221 | 39563 | 40817 |
| Pvi_95212R | GCTCCCCTAGCTTTCGTCTC | - | 92006 | 96742 | 91759 | 95212 |
|  |  | + | 123093 | 127727 | 123116 | 126176 |
| Pvi_25894F | GCTCCGATGGAAACTAGATCA | + | 24293 | 27344 |  | 25894 |
| Pvi_108187F | GCTCTTAGGAAGCAGTGCTCA | + | 105103 | 109724 | 105188 | 108187 |
| Pvi_125409R | GCTGGATCACCTCCTTTTCA | - | 122326 | 126960 | 122349 | 125409 |
|  |  | + | 92773 | 97509 | 92526 | 95979 |
| Pvi_7136R | GCTTGCCAAACAAACGC | - |  | 8206 |  | 7136 |
| Pvi_17624F | AAAATAGAATAGAGGGAGTTTCG | + |  |  |  | 17624 |
| Pvi_14720F | AATAGTCAAGTACCCCATTTCC | + |  | 15870 |  | 14720 |
| Pvi_18757R | GCTTGGGCTGCTTTAATGGT | - | 17608 | 20018 | 17695 | 18757 |
| Pvi_25288F | GCTTTTTCAGCTATTTGTAATTCG | + | 23690 | 26738 | 24395 | 25288 |
| Pvi_31373R | GCTTTTTCCCTGACTTGCTG | - | 29730 | 32846 |  | 31373 |
| Pvi_14066F | GGAAAGATGTTTTCTTCCTCCA | + |  | 15220 |  | 14066 |
| Pvi_61245R | GGAACAGGGCCTATCACAAG | - | 60042 | 62750 | 60062 | 61245 |
| Pvi_13151F | GGAACCAAAGCTCTCTGC | + |  | 14226 | 13346 | 13151 |
| Pvi_499F | GGAACCATGCATAGCACTGA | + | 493 | 1024 | 502 | 499 |
| Pvi_36871R | GGAATATAGGCGGAAACGTC | - | 35185 | 38294 | 35644 | 36871 |
| Pvi_45138R | GGAATCAACAAGAGAAAAACTTTG | - | 43383 | 46528 | 43945 | 45138 |
| Pvi_36471F | AATATGTGTTTATGTAGCTATCGGT | + | 34785 | 37894 | 35244 | 36471 |
| Pvi_107715R | GGAATTCAATAAATAAAAGTGGATT | - |  |  |  | 107715 |
| Pvi_121518R | GGACGGAGGAGGCTAGGTTA | - | 118431 | 123068 | 118597 | 121518 |
|  |  | + | 96668 | 101401 | 96278 | 99870 |
| Pvi_7853R | GGAGAGATGGCTGAGTGGAC | - | 7897 | 8925 | 7976 | 7853 |
| Pvi_10070F | GGAGCAATGAACCTATTTGAAG | + | 10101 | 11139 | 10196 | 10070 |
| Pvi_109499R | GGAGGCTCTATTATCCAATTATTA | - |  |  |  | 109499 |
| Pvi_4721F | GGAGGTTTTCTCCTCATACGG | + | 4750 | 5273 | 4781 | 4721 |
| Pvi_70293F | GGAGTATTTCAGGAGGAACTGTAA | + | 69031 | 71755 | 68904 | 70293 |
| Pvi_48442F | GGATAGGTGCAGAGACTCAATG | + | 46737 | 49909 | 47207 | 48442 |
| Pvi_42918F | GGATCCCTATCCACAACAA | + | 41198 |  | 41664 | 42918 |
| Pvi_18889R | AATCCATAGTCTTAGGGCAGAA | - |  |  |  | 18889 |
| Pvi_80234F | GGATCTCGACAATACGAAGCA | + | 79066 | 81677 | 78865 | 80234 |
| Pvi_20920R | GGATCTTTAATTGTTGGAAACTT | - | 19322 |  | 20024 | 20920 |
| Pvi_52198F | GGATGGAATTCAAATCAGTTAAG | + |  |  |  | 52198 |
| Pvi_111245R | GGATGTAAAGCCAAGCAAAT | - | 108150 | 112750 | 108196 | 111245 |
| Pvi_15700R | GGATTCGAACCGATGACTTC | - |  |  |  | 15700 |
| Pvi_118951R | GGATTCGAACCTACGACCAG | - | 115872 | 120501 | 116040 | 118951 |
|  |  | + | 99227 | 103968 | 98835 | 102437 |
| Pvi_28007F | GGATTTGTTGGCGAAAA | + |  | 29478 |  | 28007 |
| Pvi_121185R | GGCAAAATAGCCCCGTAACT | - | 118099 | 122735 | 118264 | 121185 |
|  |  | + | 97000 | 101734 | 96611 | 100203 |
| Pvi_85057R | GGCCTCGTTGCTAAGTGCTA | - | 83907 | 86512 | 83668 | 85057 |
|  |  | + | 131192 | 137957 | 131207 | 136331 |
| Pvi_112107F | AATCGAAAATAAATAGACACTCAAA | + | 108970 | 113591 | 109048 | 112107 |
| Pvi_120253R | GGCGTTAGAGCATTGAGAGG | - | 117169 | 121803 | 117332 | 120253 |
|  |  | + | 97930 | 102666 | 97543 | 101135 |
| Pvi_67189F | GGCTCGTATTTTATCTTTCTTACCA | + | 65925 | 68649 |  | 67189 |
| Pvi_3692F | GGCTCTATCCATTTATTCACTAGAC | + |  | 4232 | 3702 | 3692 |
| Pvi_33811R | GGCTGTCTCGCAATACCTTC | - | 32147 | 35224 | 32611 | 33811 |
| Pvi_26442F | GGCTTCCATCAAGCCACTAC | + | 24862 | 27913 | 25575 | 26442 |
| Pvi_138577R | GGCTTTCTAGATGTATATGACGAT | - | 133415 | 140178 | 133429 | 138577 |
|  |  | + | 81680 | 84287 | 81442 | 82807 |
| Pvi_3568R | GGGAAAGGTTAGGTTTTCCTAT | - |  | 4095 |  | 3568 |
| Pvi_126815R | GGGAAGTGGTGTTTCCAGTG | - | 123732 | 128366 | 123755 | 126815 |
|  |  | + | 91367 | 96103 | 91120 | 94573 |
| Pvi_62126F | GGGACAAATTTCTTGTGAGC | + |  | 63621 |  | 62126 |
| Pvi_132000R | GGGAGTCAGAGTCGAAAAGAG | - | 128951 | 133618 |  | 132000 |
|  |  | + | 86147 | 90850 |  | 89387 |
| Pvi_70884F | GGGATCAGGGATACTTTCAGC | + | 69622 | 72346 | 69495 | 70884 |
| Pvi_93006R | GGGATTTTTCTTGGGAAGTTT | - | 89801 | 94491 | 89559 | 93006 |
|  |  | + | 125297 | 129977 | 125315 | 128381 |
| Pvi_38992R | GGGCGCGGTGGTACT | - |  |  |  | 38992 |
| Pvi_96960R | GGGCGCTCGTAGTGACTT | - | 93752 | 98485 |  | 96960 |
|  |  | + | 121349 | 125986 |  | 124430 |
| Pvi_123809R | GGGCTGTCAAAAAGGTAGAGG | - | 120730 | 125365 | 120895 | 123809 |
|  |  | + | 94368 | 99103 | 93979 | 97578 |
| Pvi_90444R | GGGGGAATGTTTTTATGTGG | - | 87206 | 91901 | 86968 | 90444 |
|  |  | + | 127893 | 132568 | 127907 | 130944 |
| Pvi_32184F | GGGGTTATATGATATATCGGGT | + |  | 33652 |  | 32184 |
| Pvi_115038F | GGGTAAAGAGTTTACACTGCTTATG | + |  | 116552 |  | 115038 |
| Pvi_82449R | GGGTAGTTATGAACCCTGTGGA | - | 81322 | 83929 | 81084 | 82449 |
|  |  | + | 133775 | 140538 | 133789 | 138937 |
| Pvi_91280R | GGGTCGAAATATGGCTTTCA | - | 88057 | 92750 | 87819 | 91280 |
|  |  | + | 127042 | 131719 | 127056 | 130108 |
| Pvi_5430R | AATGCTCTTGGCTCGACATAG | - | 5430 | 5985 | 5501 | 5430 |
| Pvi_72298F | GGGTCGCAATGGCTTTATT | + | 71011 | 73757 | 70892 | 72298 |
| Pvi_135950R | GGGTCGCTTCTTATGGACT | - |  | 137576 |  | 135950 |
|  |  | + |  | 86894 |  | 85439 |
| Pvi_122518R | GGGTGACCGATAGCGAAGTA | - | 119436 | 124073 | 119602 | 122518 |
|  |  | + | 95663 | 100396 | 95273 | 98870 |
| Pvi_76846F | GGGTGATGCTTTTCTTGAGC | + | 75608 | 78294 | 75419 | 76846 |
| Pvi_91224R | GGGTGCCATACGCAAA | - | 88001 | 92694 | 87763 | 91224 |
|  |  | + | 127102 | 131779 | 127116 | 130168 |
| Pvi_45587R | GGGTTGGGAATTATGCCTAGA | - | 43829 | 46969 | 44400 | 45587 |
| Pvi_122057R | GGTACCAAATCGAGGCAAAC | - | 118975 | 123612 | 119141 | 122057 |
|  |  | + | 96124 | 100857 | 95734 | 99331 |
| Pvi_15608F | GGTAGAGTAATGCCATGGTAAGG | + | 15076 | 16775 | 15052 | 15608 |
| Pvi_81835R | GGTAGGTCGCAAATTGGG | - | 80707 | 83315 | 80469 | 81835 |
|  |  | + | 134394 | 402 | 134408 | 139555 |
| Pvi_12264R | GGTCGACCGTTTCATTTTCTAT | - | 12257 | 13335 |  | 12264 |
| Pvi_63239F | AATGGAATTCCTTGTCGG | + |  | 64746 |  | 63239 |
| Pvi_101183R | GTACACCAGAGGTGCGTCCT | - | 97978 | 102714 | 97591 | 101183 |
|  |  | + | 117121 | 121755 | 117284 | 120205 |
| Pvi_130524R | GTACATGCCAGATCATGAATTA | - | 127473 | 132148 | 127487 | 130524 |
|  |  | + | 87624 | 92319 | 87386 | 90862 |
| Pvi_125431R | GTAGCCGTACTGGAAGGTG | - | 122348 | 126982 | 122371 | 125431 |
|  |  | + | 92752 | 97488 | 92505 | 95958 |
| Pvi_99809R | GTCCATCGACTACGCCTTTC | - | 96607 | 101340 | 96217 | 99809 |
|  |  | + | 118492 | 123129 | 118658 | 121579 |
| Pvi_81830R | GTCGCAAATTGGGGGAAT | - | 80702 | 83310 | 80464 | 81830 |
|  |  | + | 134399 | 407 | 134413 | 139560 |
| Pvi_39284F | GTCTTACCATGAGCAGATTGTAT | + |  | 40688 |  | 39284 |
| Pvi_56883F | GTGAAATCAAGGGGCATTA | + |  | 58390 | 55607 | 56883 |
| Pvi_139196R | GTGGATCAAGGCAGTGGATT | - | 134034 | 43 | 134048 | 139196 |
|  |  | + | 81065 | 83672 | 80827 | 82192 |
| Pvi_13690R | GTGGTAAAAGTGTGATTCGTTCT | - | 13716 | 14780 | 13879 | 13690 |
| Pvi_59219F | GTGGTTATGACCGATTCGATA | + | 57970 | 60726 | 57933 | 59219 |
| Pvi_38034R | GTTACCGGGTGCAACAAGAT | - | 36320 | 39447 | 36788 | 38034 |
| Pvi_129736R | GTTCAAGAATCCGTTTTCTTT | - | 126670 | 131347 | 126684 | 129736 |
|  |  | + | 88428 | 93121 | 88190 | 91651 |
| Pvi_98266R | GTTCACGGGCTGGAGATAAG | - | 95058 | 99791 | 94662 | 98266 |
|  |  | + | 120041 | 124678 | 120213 | 123122 |
| Pvi_101460R | GTTCGTTAGGATGCCTCAGC | - | 98254 | 102991 | 97868 | 101460 |
|  |  | + | 116845 | 121478 | 117007 | 119928 |
| Pvi_73306F | GTTTGAGGAACGTCTTGAGAT | + | 72063 |  | 71881 | 73306 |
| Pvi_9652R | TAACCAACGTTTATTGGAAAA | - | 9683 | 10721 | 9778 | 9652 |
| Pvi_78013F | TAAGCCAGGTCGGCTGATAC | + | 76842 | 79460 |  | 78013 |
| Pvi_89641R | TACGGAACCAAGGTCGAAAG | - | 86401 | 91104 | 86163 | 89641 |
|  |  | + | 128698 | 133365 | 128712 | 131747 |
| Pvi_5578F | AATGGTATCGAGAATTGATCC | + |  | 6133 |  | 5578 |
| Pvi_24852F | TAGAACCAGAATGGATGGTTT | + | 23254 | 26302 | 23959 | 24852 |
| Pvi_126720R | TAGGCTGAGGAGCAAAAGGA | - | 123637 | 128271 | 123660 | 126720 |
|  |  | + | 91462 | 96198 | 91215 | 94668 |
| Pvi_130736R | TAGGGCCGTTATGCTTATT | - | 127685 | 132360 | 127699 | 130736 |
|  |  | + | 87415 | 92110 | 87177 | 90653 |
| Pvi_132221R | TATCCGATTTGCCCTATGGA | - | 129172 | 133839 | 129187 | 132221 |
|  |  | + | 85927 | 90630 | 85688 | 89167 |
| Pvi_88291R | TATGCACCTCTTTGGGCTTC | - | 85051 | 89751 | 84812 | 88291 |
|  |  | + | 130048 | 134718 | 130063 | 133097 |
| Pvi_41549F | TATGGCGGTATCCGAAAACA | + | 39829 | 42953 |  | 41549 |
| Pvi_29987F | TCAAATTTTCATTGTTAATATGGAC | + |  | 31434 |  | 29987 |
| Pvi_88057R | TCAAGATGCCTTGATGGTGA | - | 84779 | 89511 | 84540 | 88057 |
|  |  | + | 130320 | 134958 | 130335 | 133331 |
| Pvi_110391F | TCAATCTTTCCAAATCTAATCC | + |  |  |  | 110391 |
| Pvi_88660R | TCAATGAACCCCATTCTTGC | - | 85420 | 90123 | 85181 | 88660 |
|  |  | + | 129679 | 134346 | 129694 | 132728 |
| Pvi_113447R | AATTACATCGGAGCGTTTCC | - | 110383 | 114950 |  | 113447 |
| Pvi_29252F | TCAATTCGTTCGAACTTGCTT | + | 27606 | 30693 |  | 29252 |
| Pvi_114258F | TCACAGTTCCCACCGC | + | 111189 |  | 111300 | 114258 |
| Pvi_36012R | TCACCAAGACCTATAATACGAGCA | - | 34326 | 37435 | 34785 | 36012 |
| Pvi_96104R | TCACCCTAAACGAAAGAAGA | - | 92892 | 97628 | 92645 | 96104 |
|  |  | + | 122207 | 126841 | 122230 | 125284 |
| Pvi_26609R | TCACTTGTAGCATACCATATCTCA | - |  | 28080 |  | 26609 |
| Pvi_125393R | TCAGGGAGAGCTAATGCTT | - | 122310 | 126944 | 122333 | 125393 |
|  |  | + | 92790 | 97526 | 92543 | 95996 |
| Pvi_41129R | TCAGTTGGAAAATGCAGTCG | - | 39409 | 42533 | 39875 | 41129 |
| Pvi_70413R | TCATCACAGAATATTTCTAGGTCCC | - |  | 71875 |  | 70413 |
| Pvi_124961R | TCATCCAAGGCACATTAGCA | - | 121877 | 126518 | 121903 | 124961 |
|  |  | + | 93222 | 97951 | 92972 | 96427 |
| Pvi_47846F | TCATTCGATCATTATATACATTTTG | + |  |  |  | 47846 |
| Pvi_110720F | AAAATAGATCCTAATGAAAGTCCA | + | 107629 | 112229 | 107675 | 110720 |
| Pvi_139626R | AATTCACTTAACGACGAGATTTAG | - |  |  |  | 139626 |
|  |  | + |  |  |  | 81758 |
| Pvi_101822R | TCATTCTGGCATCGAGCTATT | - | 98616 | 103358 | 98230 | 101822 |
|  |  | + | 116482 | 121110 | 116644 | 119565 |
| Pvi_63326R | TCATTGCGGGTTGGTT | - | 62228 | 64833 |  | 63326 |
| Pvi_59953R | TCCAAAGTTCCAGACATTTGTT | - | 58758 | 61469 | 58778 | 59953 |
| Pvi_80090F | TCCACATCCCTTTAGTTTTGC | + |  | 81537 | 78725 | 80090 |
| Pvi_94612R | TCCCAAGGGCAGGTTCTTAC | - | 91406 | 96142 | 91159 | 94612 |
|  |  | + | 123693 | 128327 | 123716 | 126776 |
| Pvi_88584R | TCCCTCTTACTAACTGGAATAAAA | - | 85344 | 90047 | 85105 | 88584 |
|  |  | + | 129751 | 134418 | 129766 | 132800 |
| Pvi_99849R | TCCGTACCAACAAGGGGTAG | - | 96647 | 101380 | 96257 | 99849 |
|  |  | + | 118452 | 123089 | 118618 | 121539 |
| Pvi_48276R | TCCGTAGCGTCTACCGATTT | - | 46570 | 49745 | 47041 | 48276 |
| Pvi_104115R | TCCGTATCTTATTGGTGAAACTGA | - | 100906 | 105673 | 100515 | 104115 |
|  |  | + | 114189 | 118792 | 114356 | 117269 |
| Pvi_135221R | TCCTAAACATATGGCTAGCAAC | - |  | 136849 |  | 135221 |
|  |  | + |  | 87618 |  | 86165 |
| Pvi_96848R | AATTTCACGTTCTTCCTTTCA | - | 93640 | 98373 |  | 96848 |
|  |  | + | 121458 | 126095 |  | 124539 |
| Pvi_52686R | TCCTCCTTTGAAACACTTTGG | - | 50603 | 54130 | 51392 | 52686 |
| Pvi_128457R | TCCTCTAGGTACAGCGTTTGC | - | 125378 | 130058 | 125396 | 128457 |
|  |  | + | 89720 | 94410 | 89478 | 92930 |
| Pvi_54225F | TCCTGAAGTTCTTTGTAACGTTGT | + | 52103 | 55673 | 52920 | 54225 |
| Pvi_9392R | TCGAAATATAGCTGCTACGC | - |  |  |  | 9392 |
| Pvi_52462F | TCGAACCGTAGACCTTCTCG | + | 50382 | 53910 | 51172 | 52462 |
| Pvi_6432R | TCGAAGCATTCATTTTCTTTCTC | - |  |  |  | 6432 |
| Pvi_127453R | TCGAATAGAACATGCTGAACAA | - | 124372 | 129010 | 124394 | 127453 |
|  |  | + | 90725 | 95457 | 90479 | 93933 |
| Pvi_23059R | TCGCTATCTGAGGCGTTAATTT | - | 21461 | 24509 | 22166 | 23059 |
| Pvi_124275R | TCGGAGTTATTTCCCAAGGA | - | 121194 | 125831 | 121362 | 124275 |
|  |  | + | 93905 | 98638 | 93513 | 97113 |
| Pvi_61730R | TCGTACAATTGAACCTTTTCAAAC | - | 60527 | 63235 | 60547 | 61730 |
| Pvi_54226R | ACAACGTTACAAAGAACTTCAGG | - | 52104 | 55674 | 52921 | 54226 |
| Pvi_99504R | TCTAGGCAAACCTCCTGG | - | 96302 | 101035 | 95912 | 99504 |
|  |  | + | 118799 | 123436 | 118965 | 121886 |
| Pvi_20900R | TCTCAAGTTCTTCTGCCAAGC | - | 19302 |  |  | 20900 |
| Pvi_129059R | TCTCACACCGGGTAAATCCT | - | 125993 | 130670 | 126012 | 129059 |
|  |  | + | 89106 | 93799 | 88863 | 92329 |
| Pvi_137891R | TCTCACCATCCCCATAGTGT | - | 132729 | 139492 | 132743 | 137891 |
|  |  | + | 82370 | 84977 | 82132 | 83497 |
| Pvi_96621R | TCTCCATACACTGATAAGGGAT | - | 93414 | 98146 | 93166 | 96621 |
|  |  | + | 121683 | 126321 | 121707 | 124765 |
| Pvi_107439F | TCTCCTTTTAAATGGCAGTTC | + |  |  |  | 107439 |
| Pvi_87694R | TCTCGCGTAAAGCCTTTT | - | 84411 | 89148 | 84172 | 87694 |
|  |  | + | 130690 | 135323 | 130705 | 133696 |
| Pvi_67341R | TCTTAAATTCTGGTTGGAGTTTC | - |  | 68801 |  | 67341 |
| Pvi_14862R | TCTTATCTGTTTTGGTGAGAGATT | - |  | 16005 |  | 14862 |
| Pvi_72170R | TCTTCAACGGTTTGTGTAGCC | - | 70883 | 73629 | 70764 | 72170 |
| Pvi_112477R | ACAAGATCAAACCAAATAGTAGAACA | - | 109354 | 113980 | 109435 | 112477 |
| Pvi_128021R | TCTTCCGTGATGAACTGTCG | - | 124938 | 129568 | 124960 | 128021 |
|  |  | + | 90161 | 94901 | 89915 | 93367 |
| Pvi_71818F | TCTTCTTCCGAGAACCACCT | + | 70551 | 73279 | 70423 | 71818 |
| Pvi_34563R | TCTTGAAATAGACAACTCGCACA | - | 32885 | 35985 | 33346 | 34563 |
| Pvi_85913R | TCTTGCACCTCCTCCTTT | - |  |  |  | 85913 |
|  |  | + |  |  |  | 135477 |
| Pvi_134075R | TCTTGTGGTTTCCTCGGTTT | - | 131070 | 135703 | 131085 | 134075 |
|  |  | + | 84029 | 88766 | 83790 | 87313 |
| Pvi_24233R | TCTTTCGCAAAACAATCCATC | - | 22635 | 25683 | 23340 | 24233 |
| Pvi_135747R | TCTTTGATTAGGGATTGCTATATG | - |  |  |  | 135747 |
|  |  | + |  |  |  | 85637 |
| Pvi_49480R | TCTTTGGAATAGTGGCATAAAA | - |  |  |  | 49480 |
| Pvi_58952F | TGAATTGGCGATCAGAACAC | + | 57703 | 60459 | 57666 | 58952 |
| Pvi_35890F | ACACTAATGGCAACCCTTCG | + | 34204 | 37313 | 34663 | 35890 |
| Pvi_65272F | TGACATAAGATATATGGAATCACG | + |  |  |  | 65272 |
| Pvi_129756R | TGACATGAGCGTGAAAGAGG | - | 126690 | 131367 | 126704 | 129756 |
|  |  | + | 88409 | 93102 | 88171 | 91632 |
| Pvi_110099F | TGACCAAGAGAAGTTGAAGC | + |  | 111608 | 107054 | 110099 |
| Pvi_93455R | TGACCTCTGTGCTTCTCTCAT | - | 90249 | 94989 | 90004 | 93455 |
|  |  | + | 124849 | 129479 | 124870 | 127932 |
| Pvi_80924F | TGACGTGGATTGTATCGATTTC | + | 79772 | 82400 |  | 80924 |
| Pvi_89652R | TGACTTGCTCCTACGGAACC | - | 86412 | 91115 | 86174 | 89652 |
|  |  | + | 128687 | 133354 | 128701 | 131736 |
| Pvi_23805R | TGAGCAACACCAAATCCTTCT | - | 22207 | 25255 | 22912 | 23805 |
| Pvi_23499F | TGAGCAAGAGGCTTCAAGAAA | + | 21901 | 24949 | 22606 | 23499 |
| Pvi_117020R | TGAGCCTCGTTTTGAGGAAT | - | 113939 | 118543 |  | 117020 |
|  |  | + | 101160 | 105926 |  | 104368 |
| Pvi_25206R | ACAGAACGCCCCGAGTAAT | - | 23608 | 26656 | 24313 | 25206 |
| Pvi_65915F | TGATCATATATGTATTCCAATACGG | + |  | 67337 |  | 65915 |
| Pvi_11894R | TGCAAAAACAGCTAATTGGAA | - | 11949 | 12966 | 12030 | 11894 |
| Pvi_76796F | TGCAAACCCACTGTACGA | + | 75558 | 78244 |  | 76796 |
| Pvi_27744R | TGCACATACTTACTGACAATATCCA | - | 26164 | 29215 | 26877 | 27744 |
| Pvi_85027R | TGCACTGGAACTCGTGGTTA | - | 83877 | 86482 | 83638 | 85027 |
|  |  | + | 131222 | 137987 | 131237 | 136361 |
| Pvi_139593R | TGCACTTTCATAACTCGTGAAAT | - | 134432 |  |  | 139593 |
|  |  | + | 80664 |  |  | 81792 |
| Pvi_61628F | TGCAGAAATAGTGCTTCAGGA | + | 60425 | 63133 |  | 61628 |
| Pvi_50280R | TGCAGCAGGGTTGGTTATCT | - | 48451 | 51753 | 49035 | 50280 |
| Pvi_92203R | TGCCATGTGAATCGCTAGAA | - | 88980 | 93673 | 88737 | 92203 |
|  |  | + | 126119 | 130796 | 126138 | 129185 |
| Pvi_97593R | TGCCCATCCTACCTCCTCTA | - | 94383 | 99118 | 93994 | 97593 |
|  |  | + | 120716 | 125351 | 120881 | 123795 |
| Pvi_55203F | ACAGCTCTAACTCGATTATTTCCT | + | 53081 | 56651 | 53898 | 55203 |
| Pvi_39816R | TGCCGGTCATATGTATCGAA | - | 38096 | 41220 |  | 39816 |
| Pvi_16206R | TGCCTCCTTGAAAGAGAGATG | - | 15675 | 17380 | 15506 | 16206 |
| Pvi_133399R | TGCCTTGAAGAGGACTCGAA | - | 130388 | 135026 | 130403 | 133399 |
|  |  | + | 84711 | 89443 | 84472 | 87989 |
| Pvi_111187R | TGCGAATCCGCTTGC | - |  |  |  | 111187 |
| Pvi_139545R | TGCGACCTACCATAGGATTTG | - | 134384 | 392 | 134398 | 139545 |
|  |  | + | 80714 | 83322 | 80476 | 81842 |
| Pvi_59279R | TGCGACGTTTTATTCCAG | - |  | 60786 |  | 59279 |
| Pvi_47403F | TGCGATGCTCTAACCTCTGA | + | 45757 | 48906 | 46386 | 47403 |
| Pvi_12984R | TGCGGGTTCGATTCC | - | 49099 | 14059 | 49733 | 12984 |
| Pvi_119602R | TGCGGTGACGATACTGTAGG | - | 116519 | 121147 | 116681 | 119602 |
|  |  | + | 98580 | 103322 | 98194 | 101786 |
| Pvi_44588F | TGCTCTCCATTGCTTGTGTG | + | 42830 | 45951 | 43390 | 44588 |
| Pvi_70047R | ACCCATGGAAATACCCCTTT | - | 68785 | 71509 | 68658 | 70047 |
| Pvi_5429R | TGCTCTTGGCTCGACATAGT | - | 5429 | 5984 | 5500 | 5429 |
| Pvi_3004F | TGCTGATACATTCGAATAATTAAA | + |  | 3539 | 3013 | 3004 |
| Pvi_80234R | TGCTTCGTATTGTCGAGATCC | - | 79066 | 81677 | 78865 | 80234 |
| Pvi_98760R | TGCTTTCGGCTACTGGACTT | - | 95553 | 100286 | 95163 | 98760 |
|  |  | + | 119546 | 124183 | 119712 | 122628 |
| Pvi_69550F | TGGAACACCAATGGGCATA | + | 68271 | 71008 | 68160 | 69550 |
| Pvi_29456R | TGGAATAAAAGGAATTGGTATTG | - | 27810 | 30897 |  | 29456 |
| Pvi_49871R | TGGATAGGCTGGCCCTTAC | - | 48042 | 51344 | 48626 | 49871 |
| Pvi_7487F | TGGATCTGTGTGGTAAAGAAAA | + |  |  |  | 7487 |
| Pvi_138541R | TGGATCTTATATGAATCGTATGATG | - | 133379 | 140142 |  | 138541 |
|  |  | + | 81715 | 84322 |  | 82842 |
| Pvi_76331F | TGGATTCTTCAATACCTACTATTGT | + |  | 77776 |  | 76331 |
| Pvi_99474R | ACCCCCACCTCCTTTATCAC | - | 96272 | 101005 | 95882 | 99474 |
|  |  | + | 118827 | 123464 | 118993 | 121914 |
| Pvi_87908R | TGGCGCTCTCCTCTATCTTC | - | 84630 | 89362 | 84391 | 87908 |
|  |  | + | 130469 | 135107 | 130484 | 133480 |
| Pvi_120792R | TGGCTTTGGGCCTTT | - | 117707 |  | 117871 | 120792 |
|  |  | + | 97397 |  | 97009 | 100601 |
| Pvi_133811R | TGGGATCGTTTGATCGATTT | - | 130805 | 135438 | 130820 | 133811 |
|  |  | + | 84294 | 89031 | 84055 | 87577 |
| Pvi_124882R | TGGGGCGATTCAGGT | - | 121798 | 126439 | 121824 | 124882 |
|  |  | + | 93306 | 98035 | 93056 | 96511 |
| Pvi_97818R | TGGGGTGATCTCGTAGTTCC | - | 94609 | 99343 | 94219 | 97818 |
|  |  | + | 120490 | 125126 | 120656 | 123570 |
| Pvi_79014R | TGGGTAGCCGTTGTTAAACC | - | 77844 | 80461 |  | 79014 |
| Pvi_947R | TGGTATTCGTGAGCCTGTTTC | - | 941 | 1472 | 950 | 947 |
| Pvi_49145R | TGGTGACACGAGGATTTTCA | - | 47457 | 50663 | 48088 | 49145 |
| Pvi_116757R | TGTAACACGGTGGGATTATT | - |  |  |  | 116757 |
| Pvi_57466F | TGTACAAGCTCGTAACGAAGG | + | 55375 | 58973 | 56190 | 57466 |
| Pvi_30342R | ACCCCTTGGGATCGATAAAC | - | 28723 | 31789 | 29361 | 30342 |
| Pvi_3286R | TGTATTATGCAACACCTTGTTC | - |  | 3817 |  | 3286 |
| Pvi_121771R | TGTCAAAATGCATCGGTAGG | - | 118684 | 123321 | 118850 | 121771 |
|  |  | + | 96415 | 101148 | 96025 | 99617 |
| Pvi_31653R | TGTCAGGCAACCTCGTCATA | - | 30010 | 33126 |  | 31653 |
| Pvi_39795F | TGTGCCCAATTCCGAAGTTA | + | 38075 | 41199 | 38541 | 39795 |
| Pvi_34563F | TGTGCGAGTTGTCTATTTCAAGA | + | 32885 | 35985 | 33346 | 34563 |
| Pvi_41797F | TGTGGTGTGTGAACAAGGAA | + | 40077 | 43201 | 40543 | 41797 |
| Pvi_60217F | TGTTTGGCTATTCTAAGTGGTTCTT | + | 59022 | 61733 | 59042 | 60217 |
| Pvi_66368F | AAAATGGAAGAATAAAAGAATAGGT | + |  |  |  | 66368 |
| Pvi_83908R | ACCCGGAAAGGGTAGAAGAA | - | 82781 | 85388 | 82543 | 83908 |
|  |  | + | 132318 | 198659 | 132332 | 195461 |
| Pvi_8579F | TTAAAAGATAGGCTTTGAAATAGG | + |  |  |  | 8579 |
| Pvi_67339R | TTAAATTCTGGTTGGAGTTTCTT | - |  | 68799 |  | 67339 |
| Pvi_114037F | TTAATACCAGAATATTCGGTTTG | + |  | 115540 |  | 114037 |
| Pvi_46181R | TTAATTTGATACATTGTGATTGCT | - |  |  |  | 46181 |
| Pvi_84042R | TTACAGAAAAGAGTCTTCGTTTATT | - |  | 85522 |  | 84042 |
|  |  | + |  | 198381 |  | 195266 |
| Pvi_115989F | TTACATAAAGCTCTTGCTTTGATA | + |  | 117512 |  | 115989 |
| Pvi_21860R | TTACCAAAGTTTGTGGAGTCG | - | 20262 | 23310 |  | 21860 |
| Pvi_14329F | TTACTAATTTCCTCTCTTCTTTTCC | + |  |  |  | 14329 |
| Pvi_55204R | TTAGGAAATAATCGAGTTAGAGCTG | - | 53082 | 56652 | 53899 | 55204 |
| Pvi_92630R | TTAGTCTTCGGGACGGAGTG | - | 89408 | 94100 | 89163 | 92630 |
|  |  | + | 125691 | 130369 | 125712 | 128758 |
| Pvi_52464R | ACCGAGAAGGTCTACGGTTC | - | 50384 | 53912 | 51174 | 52464 |
| Pvi_47810F | TTATAATAAAGAGAAAATGCCAAGA | + |  |  |  | 47810 |
| Pvi_87492R | TTATTCGTTCTTTGAGCAAAA | - | 84208 | 88945 | 83969 | 87492 |
|  |  | + | 130890 | 135523 | 130905 | 133895 |
| Pvi_32809F | TTATTTACAAGCGGTATTCAAGC | + | 31161 | 34277 | 31848 | 32809 |
| Pvi_131754R | TTCAATCCTTTCGACCTTGG | - | 128705 | 133372 | 128719 | 131754 |
|  |  | + | 86394 | 91097 | 86156 | 89634 |
| Pvi_74664F | TTCAATGAGTAGTTATAGGCGCA | + |  | 76108 |  | 74664 |
| Pvi_82197R | TTCACAATCCACTGCCTTGA | - | 81070 | 83677 | 80832 | 82197 |
|  |  | + | 134029 | 38 | 134043 | 139191 |
| Pvi_118236R | TTCACAGGTATCACTTTTCACGA | - | 115146 | 119763 | 115308 | 118236 |
|  |  | + | 99950 | 104703 | 99564 | 103149 |
| Pvi_35329F | TTCATGTTTGGTTCGGGAAG | + | 33648 | 36759 | 34109 | 35329 |
| Pvi_132962R | TTCCATAGGCTCGAATGACC | - | 129913 | 134580 | 129928 | 132962 |
|  |  | + | 85186 | 89889 | 84947 | 88426 |
| Pvi_34488F | ACCGATATTTTAGCAACAAATCTAA | + | 32810 | 35910 | 33271 | 34488 |
| Pvi_114051F | TTCGGTTTGATAACCTGCTA | + | 110982 | 115554 |  | 114051 |
| Pvi_54587R | TTCGTTTTGTTCAAGCAGGA | - | 52465 | 56035 | 53282 | 54587 |
| Pvi_112337F | TTCTAAGTATTTCTTATTGTCGAGC | + |  | 113840 |  | 112337 |
| Pvi_128056R | TTCTATTAGTTAGCGATCCCG | - | 124973 | 129603 |  | 128056 |
|  |  | + | 90125 | 94865 |  | 93331 |
| Pvi_56135R | TTCTCACAACGACAGGGTCT | - | 54044 | 57642 |  | 56135 |
| Pvi_136860R | TTCTCGCTATTTGGAATGGA | - | 131715 | 138486 | 131730 | 136860 |
|  |  | + | 83384 | 85983 | 83145 | 84528 |
| Pvi_19456F | TTCTGACATCTTAGTCTCGTAGTTT | + |  | 20726 |  | 19456 |
| Pvi_62668R | TTCTGGATTGGGTTCATCTCT | - | 61569 | 64175 | 61408 | 62668 |
| Pvi_92518R | TTCTGTAGAGGGACAGTAAGGAT | - |  | 93988 |  | 92518 |
|  |  | + |  | 130478 |  | 128867 |
| Pvi_25382R | TTCTTGAAGTATTTCCCATACAAT | - | 23784 | 26832 | 24489 | 25382 |
| Pvi_7215R | TTCTTGGCTCTTATCCATTTT | - |  |  |  | 7215 |
| Pvi_608F | TTGAAAGTACCAGATATTCCTAAAG | + | 602 | 1133 |  | 608 |
| Pvi_134229R | TTGACAAATCGGGTGTCATA | - |  | 135857 |  | 134229 |
|  |  | + |  | 88612 |  | 87159 |
| Pvi_59835R | TTGAGTTGTTAATGAATCTGCTT | - |  | 61351 |  | 59835 |
| Pvi_112075R | TTGATCACAAGCCGAAACAT | - | 108938 | 113559 | 109016 | 112075 |
| Pvi_132730R | TTGCAAGAATGGGGTTCATT | - | 129681 | 134348 | 129696 | 132730 |
|  |  | + | 85418 | 90121 | 85179 | 88658 |
| Pvi_22358F | TTGCATGGGAACAGATTCAT | + | 20760 | 23808 | 21465 | 22358 |
| Pvi_133180R | TTGCATTATCCGTAGGACACC | - | 130119 | 134801 | 130134 | 133180 |
|  |  | + | 84979 | 89667 | 84740 | 88207 |
| Pvi_136514R | TTGGTCAAATACCTAACGAAA | - |  | 138140 |  | 136514 |
|  |  | + |  | 86328 |  | 84873 |
| Pvi_30641F | TTGGTTGAAAGGCCTGAAAG | + | 29022 | 32088 |  | 30641 |
| Pvi_127158R | ACCTTGACGTGGTGGAAGTC | - | 124076 | 128709 | 124098 | 127158 |
|  |  | + | 91023 | 95760 | 90777 | 94230 |
| Pvi_60385R | TTGTAGACCCAACGAATTAGTAG | - |  | 61901 |  | 60385 |
| Pvi_107400R | TTGTATGTGAAAGACATCTATTGC | - | 104302 | 108944 | 104208 | 107400 |
| Pvi_94431R | TTGTCTTTCCTTCCAAGGAT | - | 91225 | 95961 | 90978 | 94431 |
|  |  | + | 123874 | 128508 | 123897 | 126957 |
| Pvi_2508F | TTGTTTACGAAGAAACAATAAGAA | + |  |  |  | 2508 |
| Pvi_4463R | TTTAAAGATAAAGAACGAACTTTGA | - |  |  |  | 4463 |
| Pvi_114425R | TTTATTCTAGCAGACGGAATTG | - |  |  |  | 114425 |
| Pvi_11893F | TTTCCAATTAGCTGTTTTTGCAT | + | 11948 | 12965 | 12029 | 11893 |
| Pvi_79251F | TTTCCCTTCATTCTTCCTCTATG | + | 78081 | 80698 |  | 79251 |
| Pvi_41797R | TTTCCTTGTTCACACACCACA | - | 40077 | 43201 | 40543 | 41797 |
| Pvi_131746R | TTTCGACCTTGGTTCCGTAG | - | 128697 | 133364 | 128711 | 131746 |
|  |  | + | 86402 | 91105 | 86164 | 89642 |
| Pvi_12822R | ACCTTGAGGTCACGGGTTC | - | 12856 | 13893 | 13020 | 12822 |
| Pvi_111787F | TTTCTATTCATCTTAATGTTCCG | + |  |  |  | 111787 |
| Pvi_75795F | TTTCTTTAGATTCGTGAATCTTTT | + |  |  |  | 75795 |
| Pvi_5091F | TTTCTTTCAAAAGAGTAGCAACA | + | 5070 | 5643 | 5141 | 5091 |
| Pvi_63964F | TTTGAATCCAACTTTTCCCTTTA | + |  |  |  | 63964 |
| Pvi_43273F | TTTGAGGGTATAAGCCCCTA | + |  |  |  | 43273 |
| Pvi_13081F | TTTGATTTCATTCGGCTCCT | + | 13111 | 14156 | 13276 | 13081 |
| Pvi_61817R | TTTGCATGAGATAGAAATAAGGA | - |  | 63323 |  | 61817 |
| Pvi_70511F | TTTGGCTTTGGTGCATT | + |  | 71973 |  | 70511 |
| Pvi_114630F | TTTGTATTGCATTCCATATCTTT | + | 111569 |  |  | 114630 |
| Pvi_120913R | ACGAAAGGCGTAACGATCTG | - | 117828 | 122463 | 117992 | 120913 |
|  |  | + | 97271 | 102006 | 96883 | 100475 |
| Pvi_74524F | TTTGTTTGGATGTAAGAAATTTG | + |  |  |  | 74524 |
| Pvi_13046F | TTTTAACGTCTCTAATTCAAAACC | + |  |  | 13241 | 13046 |
| Pvi_3482F | TTTTACACATTCAATTCATAACATC | + |  |  |  | 3482 |
| Pvi_46254R | TTTTACATACCACAGTAATTTGCTA | - |  | 47754 | 45248 | 46254 |
| Pvi_110557R | TTTTATCATGTGGGAGTTAGAATTA | - | 107466 | 112066 | 107512 | 110557 |
| Pvi_43332F | TTTTATGCGATTCCAAATTC | + |  |  |  | 43332 |
| Pvi_42529R | TTTTCAGATTTGGCGAGCAT | - | 40809 | 43933 |  | 42529 |
| Pvi_96667R | TTTTCCCATTGAGGACGAAC | - | 93460 | 98192 | 93212 | 96667 |
|  |  | + | 121639 | 126277 | 121663 | 124721 |
| Pvi_125132R | TTTTCTCGCTTTTGGCGTAG | - | 122048 | 126689 | 122074 | 125132 |
|  |  | + | 93051 | 97780 | 92801 | 96256 |
| Pvi_16032R | TTTTCTGTTATGCTTTCATAGGA | - |  |  |  | 16032 |
| Pvi_31365F | TTTTGATGCAGCAAGTCAGG | + | 29722 | 32838 | 30387 | 31365 |
| Pvi_65179F | TTTTGATTGACCTCCTCTCTT | + |  |  |  | 65179 |
| Pvi_46816F | TTTTGCTTTTCCAGCGA | + | 45188 |  | 45815 | 46816 |
| Pvi_109747F | TTTTGGCATTAACATAAATTTTG | + |  |  |  | 109747 |
| Pvi_107796F | TTTTGGTATATTGTGTCCTCAGT | + |  | 109339 |  | 107796 |
| Pvi_6509F | TTTTGTGTCGTGCAGAGC | + |  |  |  | 6509 |
| Pvi_46123F | TTTTGTTTACCGAGGGTTCG | + | 44489 | 47623 | 45090 | 46123 |
| Pvi_98977R | ACGCGGTCAGAGATCACTTT | - | 95770 | 100503 | 95380 | 98977 |
|  |  | + | 119329 | 123966 | 119495 | 122411 |
| Pvi_105880F | ACGTGAAGAGGGAATTGTGC | + | 102895 |  | 102597 | 105880 |
| Pvi_32746R | AAAGAAACAAGAACAACAACGAC | - | 31098 | 34214 | 31785 | 32746 |
| Pvi_87425R | ACTATGCCAGCCCAAATCAT | - | 84141 | 88878 | 83902 | 87425 |
|  |  | + | 130958 | 135591 | 130973 | 133963 |
| Pvi_11523F | ACTCAGCCATCTCTCCACAG | + | 11575 | 12595 | 11645 | 11523 |
| Pvi_67842F | AGAAGAAACAAAAGCTCTTCTTACT | + |  | 69301 |  | 67842 |
| Pvi_120544R | AGAAGGGAGCTTGACTGCAA | - | 117460 | 122094 | 117623 | 120544 |
|  |  | + | 97639 | 102375 | 97252 | 100844 |
| Pvi_120053R | AGACAGCGACGGGTTC | - | 116970 | 121603 | 117132 | 120053 |
|  |  | + | 98133 | 102870 | 97747 | 101339 |
| Pvi_54732F | AGAGCTACTTTTGATTCCTCAATA | + |  |  |  | 54732 |
| Pvi_8260R | AGAGGCATTATTCAAGGGGTC | - |  |  |  | 8260 |
| Pvi_138544R | AGATGGATCTTATATGAATCGTATG | - | 133382 | 140145 |  | 138544 |
|  |  | + | 81712 | 84319 |  | 82839 |
| Pvi_50130F | AGCACATTGGGAACGTAAAT | + | 48301 | 51603 |  | 50130 |
| Pvi_59052R | AGCCCCAACCGCTAAGAAT | - | 57803 | 60559 |  | 59052 |
| Pvi_99918R | AAAGCAGGGTCACCTTGT | - | 96716 | 101449 | 96326 | 99918 |
|  |  | + | 118385 | 123022 | 118551 | 121472 |
| Pvi_90122R | AGCCCCTTTTCATCAATGG | - | 86884 | 91579 | 86646 | 90122 |
|  |  | + | 128216 | 132891 | 128230 | 131267 |
| Pvi_12997R | AGCCTTCCAAGCTAACGATG | - | 13027 | 14072 | 13192 | 12997 |
| Pvi_118193R | AGCGATTTTCGAACCATTTC | - | 115109 | 119720 | 115271 | 118193 |
|  |  | + | 99990 | 104749 | 99604 | 103195 |
| Pvi_107016F | AGGAAATTCTCAAATATTGCTTAC | + |  |  |  | 107016 |
| Pvi_131311R | AGGAATTTGTCGAACGAACC | - | 128260 | 132935 | 128274 | 131311 |
|  |  | + | 86839 | 91534 | 86601 | 90077 |
| Pvi_123792R | AGGAGGTAGGATGGGCAGTT | - | 120713 | 125348 | 120878 | 123792 |
|  |  | + | 94386 | 99121 | 93997 | 97596 |
| Pvi_136714R | AGGATCTAATGAATTATGGGTTTA | - |  |  |  | 136714 |
|  |  | + |  |  |  | 84670 |
| Pvi_110707R | AGGATCTATTTTATTGACAGGATTT | - | 107616 | 112216 | 107662 | 110707 |
| Pvi_113781R | AGGATGAGAATGGATCAATTATTA | - |  |  |  | 113781 |
| Pvi_37015F | AAAGCCATGAAACAAGTAGCTG | + | 35329 | 38438 | 35788 | 37015 |
| Pvi_70049F | AGGGGTATTTCCATGGGTTT | + | 68787 | 71511 | 68660 | 70049 |
| Pvi_100842R | AGTCAAGCTCCCTTCTGC | - | 97637 | 102373 | 97250 | 100842 |
|  |  | + | 117464 | 122098 | 117627 | 120548 |
| Pvi_92848R | AGTCGAAGATAGAAGAGCCC | - | 89636 | 94328 | 89396 | 92848 |
|  |  | + | 125463 | 130141 | 125479 | 128540 |
| Pvi_130176R | AGTCGCTTCCTTTTTGCGTA | - | 127110 | 131787 | 127124 | 130176 |
|  |  | + | 87989 | 92682 | 87751 | 91212 |
| Pvi_93085R | AGTTAGAAAATGGATTCCTATTTG | - | 89880 | 94570 | 89638 | 93085 |
|  |  | + | 125215 | 129895 | 125233 | 128299 |
| Pvi_5164F | ATAAATCCAATATGAACGATTGA | + |  |  |  | 5164 |
| Pvi_38591R | ATAAGCCCGTGGCTCTTTC | - | 36871 | 39995 | 37337 | 38591 |
| Pvi_42437F | ATAATGGAACCAACCAGCAAA | + |  |  | 41183 | 42437 |
| Pvi_136664R | ATATTCCTTGCTCATTATCAGACA | - |  | 138290 |  | 136664 |
|  |  | + |  | 86175 |  | 84720 |
| Pvi_1037F | ATCATCAAAACACCGAACCA | + | 1031 | 1562 | 1040 | 1037 |

^a^Primer sequences listed with both a “+” and “-” strand position are located in the inverted repeat region.

^b^Chloroplast genome positions listed for: *Oryza sativa*, *Osa*; *Sorghum bicolor*, *Sbi*; *Triticum aestivum* *Tae,* and *Panicum virgatum, Pvi.* Primers that do not have 100% sequence identity to a given chloroplast genome do not have positions listed for that genome. Primers with positions listed only under *Pvi* are finishing primers chosen using consed's autofinishing function.
