# Supplementary material for: Chloroplast Genome Variation in Upland and Lowland Switchgrass
Source: PLoS One. 2011 Aug 24;6(8):e23980. doi: 10.1371/journal.pone.0023980 (PMC3161095; doi:10.1371/journal.pone.0023980)
Supplement: Table S3 — Primers used to amplify inverted repeat (IR) region. (DOC) [file pone.0023980.s003.doc]

Table S3. Primers used to amplify inverted repeat (IR) region.

| **REGION** | **PCR product** | **Primer Sequence 5'-3'** |
| --- | --- | --- |
| IR_a_ | A1 | TGACGTGGATTGTATCGATTTC |
|  |  | AACTTGCTATCCTCTTGCCTAA |
|  | A2 | CAAACATATGCGGATCAAAT |
|  |  | CCTCTTGCTTGCTTCTGGTC |
| IR_b_ | B1 | CGTACTAAATAGCGAGCTAATGA |
|  |  | CATGGTAGCCTGCTCCAGTC |
|  | B2 | AACTTGCTATCCTCTTGCCTAA |
|  |  | GCTATGCATGGTTCCTTGGT |
